# Meta-Regression to Develop Predictive Equations for Urinary Nitrogen Excretion of Lactating Dairy Cows

**DOI:** 10.3390/ani13040620

**Published:** 2023-02-10

**Authors:** Matthew Beck, Cameron Marshall, Konagh Garrett, Terra Campbell, Andrew Foote, Ronaldo Vibart, David Pacheco, Pablo Gregorini

**Affiliations:** 1 Livestock Nutrient Management Research Unit, The Agricultural Research Service, The United States Department of Agriculture (USDA-ARS), Bushland, TX 79012, USA; 2 Faculty of Agriculture and Life Sciences, Lincoln University, Lincoln 7647, New Zealand; 3 Department of Animal and Food Sciences, Oklahoma State University, Stillwater, OK 74048, USA; 4 Grasslands Research Centre, AgResearch Ltd., Palmerston North 4442, New Zealand

**Keywords:** environmental modeling, environmental impacts, dairy cows, urine nitrogen excretion

## Abstract

**Simple Summary:**

Urinary nitrogen excretion is a significant environmental pollutant in both confined and pasture-based dairy systems. Urinary nitrogen excretion is difficult to measure, as it requires significant labor, animal manipulation, and specialized facilities. Accordingly, there is a need for accurate and precise models to predict urinary nitrogen excretion. Providing accurate and precise models to predict urinary nitrogen from milk urinary nitrogen is especially needed because policy makers are interested in limiting nitrogen emissions from dairy farms and producers need tools to make informed management decisions. We demonstrated that previously established equations had significant biases and poor predictive capability for dairy cows fed fresh forages. We established a new set of predictive equations based on milk urea nitrogen concentration, dry matter intake, crude protein content, and body weight. The developed equations showed improved agreement and predictive capabilities relative to previously published equations for both total mixed ration and fresh forage-fed cows.

**Abstract:**

Dairy cows’ urinary nitrogen (N) excretion (UN; g/d) represents a significant environmental concern due to their contribution to nitrate leaching, nitrous oxide (a potent greenhouse gas), and ammonia emissions (contributor to N deposition). The first objective of the current study was to determine the adequacy of existing models to predict UN from total mixed ration (TMR)-fed and fresh forage (FF)-fed cows. Next, we aimed to develop equations to predict UN based on animal factors [milk urea nitrogen (MUN; mg/dL) and body weight (BW, kg)] and to explore how these equations are improved when dietary factors, such as diet type, dry matter intake (DMI), or dietary characteristics [neutral detergent fiber (NDF) and crude protein (CP) content], are considered. A dataset was obtained from 51 published experiments composed of 174 treatment means. The whole dataset was used to evaluate the mean and linear biases of three existing equations including diet type as an interaction term; all models had significant linear and mean biases and two of the three models had poor predictive capabilities as indicated by their large relative prediction error (RPE; root mean square error of prediction as a percent of the observed mean). Next, the complete data set was split into training and test sets, which were used to develop and to evaluate new models, respectively. The first model included MUN and BW, and there was a significant interaction between diet type and the coefficients. This model had the worst 1:1 agreement [Lin’s concordance correlation coefficient (CCC) = 0.50] and largest RPE (24.7%). Models that included both animal and dietary factors performed the best, and when included in the model, the effect of diet type was no longer significant (*p* > 0.10). These models all had very good agreement (CCC ≥ 0.86) and relatively low RPE (≤13.1%). This meta-analysis developed precise and accurate equations to predict UN from dairy cows in both confined and pasture-based systems.

## 1. Introduction

Urinary N excretion by dairy cows represents a significant source of environmental pollution globally, in both confinement and pasture-based dairy systems [1]. Compared with other livestock systems, dairy production often has poor nitrogen (N) use efficiencies, defined as the proportion of dietary N that is captured in the product, which rarely exceeds 40%. This means that as much as 60% or more of ingested N is excreted into the environment, with a majority of the N lost to the environment in the form of urinary nitrogen (UN) [2,3]. A large proportion of excreted N is lost to the atmosphere when urinary urea is hydrolyzed by microbial ureases, such as those found in the feces [4]. In confinement feeding systems, such as in the United States, most of this excreted UN is captured in effluent ponds often with strict regulations around the management of manure to prevent nutrient leaching into groundwater [5]. However, in intensively managed temperate pastoral systems, such as those found in New Zealand, Ireland and parts of South America, approximately 82% of UN is discharged onto pastures [6,7]. Due to high N loading rates (equivalent to 200–2000 kg UN/ha) at the urine patch, which outpace the ability of sward plants to utilize N, the proportion of UN excreted onto pastures that is leached depends on many factors (e.g., amount of UN applied, season, drainage, soil characteristics, etc.), with a range of 1–76% being reported [8]. When considering the environmental impacts, it is necessary to conduct research aimed at improving N use efficiency and reducing UN excretion to reduce unnecessary inefficiencies, but also, it is important to quantify UN excretion in commercial settings. Yet, directly measuring UN excretion is laborious, requires specialized facilities, and requires significant animal handling and restraint, with consequent animal welfare considerations. Accordingly, accurate and precise methods to predict UN excretion are required.

Urea is produced in the liver to remove ammonia (NH_3_) from the blood stream. After ureagenesis, urea equilibrates across the different body fluids—e.g., blood, rumen, milk, and urine [4]. Some urea is recycled to the rumen from blood via saliva, and in the rumen, it is rapidly converted to NH_3_, which either is transported back across the rumen wall to re-enter the urea cycle, or utilized by rumen microorganisms. Some of the urea circulating in blood is excreted through milk or urine [4]. Urea represents around 50 to over 90% of UN, depending on the diet [9,10], and when crude protein (CP) is more than 21% of the diet DM, as frequently observed in temperate pasture-based dairy production, urinary urea N may account for as much as 96% of UN [11]. Because urea equilibrates through the different bodily fluids and it represents such a large proportion of the UN excreted, modeling efforts have been made to use blood urea N [12] and milk urea N (MUN) spot sampling [13,14,15,16] to estimate daily UN excretion. In most dairy cow production systems, MUN is especially promising as it requires no additional sampling or animal handling, as cows are already milked at least once a day [17]. Due to the ease of collection and monitoring, it has been suggested to use MUN in management and policymaking decisions to reduce N emissions from dairy farms [18].

To date, most of the modeling attempts with MUN to predict daily UN excretion has been generated using data of dairy cattle in confinement feeding operations [i.e., total mixed ration (TMR)-fed cows] [13,14,15]. There are concerns that existing equations may not be suitable to predict urinary nitrogen in pasture-based systems because they do not account for differences that are intrinsic to the two production systems, such as diet attributes. In a literature review, Spek et al. [4] reported that the relationship between MUN and urinary urea N excretion changes based on neutral detergent fiber (NDF), CP, non-protein N content of the diet, and also the NDF to starch ratio of the diet as these affect N digestion and N use efficiency. Therefore, changes in dietary characteristics (i.e., fresh forage or TMR-based diets) may mean that equations predicting UN from MUN developed from TMR-fed cows might not be applicable for fresh forage-fed cows. The possibility of UN predictive equations not being applicable to fresh forage-based dairy systems led Christodoulou et al. [19] to evaluate models of N excretion for cows fed perennial ryegrass. However, none of the models evaluated by Christodoulou et al. [19] included MUN as a predictor variable. Thus, it is necessary to evaluate available models and to develop new models for predicting UN, which include MUN as a predictor variable, for their suitability for cows fed TMR and fresh forage-based diets.

We hypothesized that existing models developed from cows fed TMR diets would have significant biases when used to predict UN from cows fed fresh forages. Thus, the first objective was to determine the applicability of established UN prediction equations [13,14,16] to excretion data from dairy cows fed fresh forages. The second objective was to establish robust equations predicting UN from MUN based on treatment means obtained from available literature and to see if this relationship was influenced by diet, i.e., TMR or forage-fed, and dietary nutritive characteristics.

## 2. Materials and Methods

### 2.1. Meta-Analysis Data Frame

**Figure 1:**
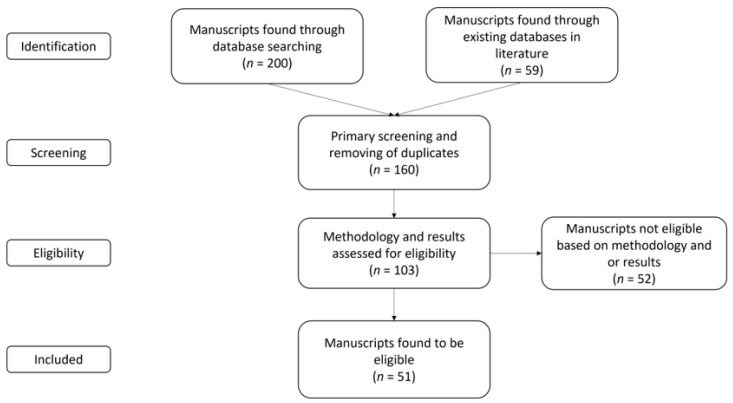
PRISMA flow diagram of the systematic review from the number of manuscripts identified by the search engine and other sources to initial screening, determining eligibility, to the final number of manuscripts included in the data frame. The n refers to the number of manuscripts.

Searches were conducted using the Google Scholar search engine with the following search terms: “nitrogen balance dairy cows”, “nitrogen partitioning dairy cows”, “fresh forage dairy cow nitrogen balance”, and “*Lolium perenne* dairy cow nitrogen balance”. Only the first 50 results of each of these search terms were read for appropriateness. The PRISMA flow diagram depicting the systematic review of the literature is displayed in Figure 1. A completed PRISMA checklist is provided in the Supplemental Materials (Table S1).

To be selected, the manuscripts must have been written in English, published in a peer-reviewed journal or a dissertation, used dairy cows as experimental animals, reported MUN content and body weight, and measured daily UN excretion (g/d) via the total collection of urine. Requiring UN excretion to be measured via total collection excluded 4 pasture-based experiments that used urine sensors (as validated by Marshall et al. [20]) to measure daily urine volume and then estimated UN excretion (g/d) based on UN concentrations of spot samples [17,21,22,23]. Furthermore, experiments must have reported MUN content and UN excretion measured during the same sampling timeframe. If the reviewed manuscript met all these parameters, they were included in the data frame.

To build on existing literature and data frames, the data frame constructed by Johnson et al. [24] was also examined for usability. Of the 59 manuscripts referenced by Johnson et al. [24], 21 experiments were incorporated into the current study. The remainder of the experiments referenced by Johnson et al. [24] were excluded. Twenty of the experiments did not report MUN, nine estimated UN (g/d) using calculations, three experiments did not adequately report dietary nutritive values, two did not report body weight, one was a duplicated citation that was listed twice, and the final two were removed because it was unclear if the subsampled animals were used to determine the reported UN excretion and MUN content of milk.

In addition to the 21 experiments used previously by Johnson et al. [24], another 30 experiments were identified from Google Scholar, resulting in a total of 51 experiments that were used to create the final data frame. The identified experiments were then grouped as either fresh forage-based experiments (*n* = 10) or TMR-based experiments (*n* = 41).

The experiments selected for the final data frame were published in the *Journal of Dairy Science* (36), *Animal Production Science* (3), *New Zealand Journal of Agricultural Research* (2), *Journal of Animal Science* (2), *Animal Feed Science and Technology* (2), *Livestock Science* (2), *Animal Science Journal* (1), *Animals* (1), *Asian-Australasian Journal of Animal Science* (1), and a thesis (1).

Once a manuscript was identified as appropriate, data were extracted and added to a data frame in Excel (v. 2211, Microsoft Corporation, Redmond, WA, USA), and visually assessed to determine if values seemed logical and biologically feasible. No data were excluded based on this assessment. No manuscripts were rejected beyond this point. Data on CP, NDF, and roughage % of the diet were evaluated and included into the data frame, as were results regarding UN and MUN content, BW, DMI, N intake, milk yield, and days in milk (DIM) from the animals used in each experiment. The complete data frame including all variables used for modeling that was compiled for this meta-analysis is presented in the Supplementary Materials (Table S2). Manuscripts within the data frame were randomly selected using a random number generated in Excel to assign the manuscripts’ data to either a training set (*n* = 38 TMR-based and *n* = 7 fresh forage-based experiments) or a test set (*n* = 3 TMR-based and 3 fresh forage-based experiments). An overview of the data frame generated from the literature is shown in Table 1. The full training set was composed of 153 treatment means, and the test set was composed of 21 treatment means. Of the selected experiments, 38 (74.5%) were a Latin-square or cross-over design, 12 (23.5%) were a completely randomized design, and 1 (2%) was an incomplete cross-over design. Accordingly, the 1964 animal observations composing the treatment means were generated from 666 cows. The experiments selected predominantly used Holstein-Friesian dairy cows, with 78% of the treatment means were derived from cows described as Friesian, Holstein, or Holstein-Friesian; 3% were from Jersey, 4% from crosses (*n* = 6 Holstein-Friesian × Jersey; *n* = 1 Holstein × Swiss Red), 6% resulted from multiple breeds composing each treatment mean, and 9% of treatment means were from other breeds (*n* = 2 were not specified; *n* = 3 were Swiss Red; *n* = 6 were Norwegian Red; *n* = 4 were Ayrshire). 

Only nine experiments reported BW for each treatment mean, and where this was available, these BWs were used in the dataset. Two experiments reported DMI as a percentage of BW, which was used to calculate BW for each treatment mean. The remainder of the experiments (40) only reported an average BW for all the cows included in their experiment. This reported mean BW was used in the dataset for this study. The authors of these 40 experiments stated that BW was used as a blocking factor when cows were randomized to treatments. Additionally, many of these experiments were a cross-over or Latin-square design, meaning that all cows were used to generate the treatment means. As such, we assumed that using the reported average BW for all cows in the dataset was valid. Nitrogen content was determined using combustion and a CN analyzer in 45% of the experiments and using the Kjeldahl method in the remaining experiments (55%). Some of the fresh forage-based experiments fed cows supplements, which can be seen by the percentage of forage of the diet (Table 1). In these instances, the CP content of their total diet was calculated based on the reported total N intake (N × 6.25 = CP) and total DMI.

In some instances, the MUN content needed to be converted to MUN in mg/dL. In order to convert from milk urea in mmol/L to MUN in mg/dL, the value was divided by 0.357. To convert from milk urea in mg/dL to MUN in mg/dL, the value was multiplied by 0.467 (urea is 46.7% N). In one instance [25], the authors were incorrect in their calculations for converting milk urea in mmol/L to MUN in mmol/L. Where 1 mmol/L of milk urea equals 2 mmol/L of MUN, they multiplied milk urea in mmol/L by 0.467. Their calculations were provided in the materials and methods, which were used to back calculate milk urea in mmol/L, which was then re-converted to MUN in mg/dL. 

### 2.2. Assessing Established Equations

Three established equations were assessed in this current study. The first equation predicted UN excretion from MUN content only:

UN (g/d) = 12.54 × MUN (mg/dL)(1)

Jonker et al. [13] developed this equation using data from three separate digestibility and N balance studies that used 10 TMR diets, 40 cows, and had 70 observations. The next equation we investigated was:

UN (g/d)/BW (kg) = 0.0259 × MUN (mg/dL) (2)

Kauffman and St-Pierre [14] obtained data from four Holstein and four Jersey cows that were involved in a split-plot Latin-square design, where they were fed TMR that contained either 13% or 17% dietary CP and 30% or 40% dietary NDF set up in a 2 × 2 factorial arrangement. The final established equation we investigated was:

UN (g/d) = −148.8 + 8.06 × MUN (mg/dL) + 8.91 × CP (%) + 4.06 × DMI (kg)(3)

This equation was developed by Spek et al. [16] from a meta-analysis that included 123 treatment mean observations. Spek et al. [16] explored how data from North America and northwestern Europe differed; however, region did not significantly influence the coefficients of Equation (3). Accordingly, the coefficients used in Equation (3) are from the northwestern Europe columns (see Table 3 of Spek et al. [16]).

The complete data set was used to assess Equations (1)–(3). First, we calculated the predicted UN output using each of the equations and then calculated the residuals (observed UN minus predicted UN). The predicted UN was then mean centered. Mixed model regression using the mean-centered predicted UN with an interaction term for diet type (TMR or fresh forage) was used as the independent variables, the residuals as the dependent variable, and the experiment as a random effect was conducted using the ‘lmer’ function of the ‘lme4’ package [26] of R [27]. Furthermore, the standard error of the mean (SEm) of UN was used as a weighting factor in the regression as suggested by St-Pierre [28]. Briefly, the optimal weight was calculated using w1/mean(w1), where w1 equals the inverse of the squared SEm. This centers the weight property around 1, thereby allowing for optimal weighting and keeping the variance and covariance components on the same scale as the dependent variable (in this case g UN per d) [28]. Mean centering the predicted UN values results in the intercept of the linear model representing the mean prediction bias and the linear slope representing the linear prediction bias, while correcting for the random effects of experiment and properly weighting the treatment means’ UN SEm [28]. The inclusion of the interaction of diet type allowed us to determine the mean and linear prediction biases for Equations (1)–(3) for both diet types. Next, we determined the root mean square error of prediction (RMSEP) and the relative prediction error (RPE; RMSEP as a % of the observed mean), for both TMR-fed and fresh forage-fed cows using the three equations, as an indication of model precision.

Finally, we regressed residuals (observed UN minus predicted UN) against variables that are commonly different between TMR-based and pastoral-based dairy systems (Table 1). Linear mixed effects modeling was again done using the ‘lmer’ function of ‘lme4’ package using the experiment as a random effect and weighting UN SEm as suggested by St-Pierre [28] and described above. The variables we considered for fixed effects were MUN, dietary CP (%), dry matter intake (DMI, kg), and body weight (BW).

### 2.3. Model Development and Evaluation

As mentioned above, the experiments were stratified by diet type and then randomly assigned to a training and a test set. This was done so that the two datasets were completely independent of each other. All modeling was done using the ‘lmer’ function of the ‘lme4’ package [26] of R [27]. All models included the unique experiment’s digital object identifier (DOI) as a random effect and UN SEm was used as a weighting factor as described above [28]. The models that we developed are based on the knowledge gained from the previously described Equations (1)–(3) [13,14,16]. As we know that BW plays a large role in the MUN to UN ratio [14], the first model developed used UN per BW as the dependent variable and MUN with diet type included as an interaction.

The next model we developed was based on the model of Spek et al. [16]. This model used UN as the dependent variable and included MUN, CP, and DMI as dependent variables, with diet type included with all possible interactions. Next, Spek et al. [16] found that BW was a significant variable in their modeling, but since it did not improve the model fit as determined by RMSEP and R^2^_,_ it was not included in their final model. However, we wanted to test this in our own data set, so the next model developed explored MUN, CP, DMI, and BW with diet type and all possible interactions as prediction variables of UN. The final model developed explored MUN, CP, and DMI and all possible interactions of diet type to predict g UN per d/kg BW. For all the models developed, if a model term was not significant, it was removed from the final model.

Once the final models were established, model evaluation was done using the test data set (Table 1). Predictions of UN were made and compared to the observed UN that was reported in the experiments. Pearson’s correlation was determined, as an indication of precision, using the ‘cor.test’ function of base R [27]. Additionally, Lin’s Concordance Correlation Coefficient (CCC) [29,30] was obtained using the ‘CCC’ function of the ‘DescTools’ package of R [31]. The CCC is a simultaneous measure of precision and accuracy and is a measure of how far the regression line of predicted and observed values deviates from the line of unity, which would indicate perfect agreement. The CCC is calculated as Pearson’s correlation multiplied by a bias correction factor (Cb), where a Cb of 1 indicates no deviation from the line of unity. As with Pearson’s correlation, CCC can range from −1 to 1, but in this instance, only values close to 1 suggest good agreement. We defined CCC values of <0 as no agreement, 0–0.20 as poor, 0.21–0.40 as weak, 0.41–0.60 as moderate, 0.61–0.80 as good, and >0.80 as excellent agreement. The root mean square error of prediction was obtained using the ‘RMSEP’ function of the ‘chillR’ package in R [32]. The RMSEP is a measure of predictive capability and has the same units as the observed and predicted values, in this instance g UN/d. The RMSEP was also expressed as a percentage of the observed mean, an estimate often referred to as relative prediction error (RPE).

## 3. Results

### 3.1. Performance of Established Equations

Table 2 reports the mean and linear prediction biases, RMSEP, and RPE of Equations (1)–(3) for the TMR and fresh forage diet types. When regressing Equation (1) mean centered predictions against observed minus predicted UN, there was a significant (*p* = 0.02) interaction for the intercept and the slope between TMR and fresh forage diet types. It was determined that Equation (1) had a significant (*p* < 0.01) mean prediction bias for the TMR diet only. This indicates that on average, UN was over predicted by 31.4 g/d for TMR-fed cows. While the mean prediction bias was not significant for pasture fed cows, there was a significant (*p* < 0.01) linear prediction bias. Furthermore, for both diet types, there was large RMSEP (>50 g UN per d) and RPE (>25% of the observed UN mean).

For Equation (2), there was only a significant (*p* < 0.01) effect of diet type on the linear prediction bias. However, the mean and linear prediction biases were not significantly different (*p* ≥ 0.15) from 0 for the fresh forage-fed cows. The mean and linear prediction biases were significantly greater than 0 for the TMR-fed cows (*p* < 0.01). There were also large RMSEP (≥49 g UN per d) and RPE (≥24.9%) for both diet types.

Equation (3) tended (*p* = 0.08) to have a significant mean prediction bias, and there was a linear prediction bias observed (*p* < 0.01) for pasture fed cows only. Equation (3) overall performed well for TMR-fed cattle, where there was no mean or linear prediction bias detected (*p* ≥ 0.23). Furthermore, Equation (3) had the smallest RMSEP and RPE, relative to the other two equations.

The residuals (observed minus predicted) of all three equations decreased with increasing MUN (Table 3). Dietary CP content only significantly influenced the residuals of Equation (3), where for every 1% unit increase in CP content, there was a −1.7 g UN/d reduction in residuals. Dry matter intake only associated to the residuals of Equation (1), where residuals increased (*p* < 0.01) by 5.6 g UN/d for every additional kg increase in DMI. Finally, the residuals increased (*p* < 0.01) with increasing BW for Equations (1) and (3) by 0.2 and 0.1 g UN/d, respectively. However, BW was not significantly associated with residuals of Equation (2).

### 3.2. Model Development

Table 4 reports the equations developed in this current study. When modeling UN (g/d) per BW (kg) as a function of MUN (mg/dL), it was determined that the intercept was not significant (*p* = 0.93), but there was a significant interaction (*p* < 0.01) of diet type and MUN. It was determined that, for every additional mg of MUN per dL of milk, there was an increase in UN (g/d) per BW (kg) of 0.0214 for TMR-fed cows and 0.0240 for pasture fed cows—a 12% increase. Both sides of the equation can be multiplied by BW without loss of generality [14], yielding the final equation:

For TMR: UN (g/d) = 0.0214 × MUN (mg/dL) × BW (kg) and 
For Pasture: UN (g/d) = 0.0240 × MUN (mg/dL) × BW (kg)(4)

When modeling UN (g/d) as a function of MUN, CP, and DMI, it was determined that none of the terms had a significant interaction with diet type (*p* ≥ 0.19) and so it was removed from the final model. This resulted in:

UN (g/d) = −209.4 + 6.8 × MUN (mg/dL) + 12.4 × CP (%) + 4.8 × DMI (kg)(5)

The next model included UN (g/d) as the dependent variable and MUN, CP, DMI, and BW as fixed effects. Similar to Equation (5), diet type was not a significant interaction for any of these variables and was therefore removed from the final model. It was determined that:

UN (g/d) = −281.3 + 6.7 × MUN (mg/dL) + 13.2 × CP (%) + 2.8 × DMI (kg) + 0.16 × BW (kg)(6)

The final model developed in this meta-analysis included UN (g/d) per BW (kg) as a function of MUN, CP, DMI, and diet type involved in all possible two-way interactions. As with Equations (5) and (6), diet type was not involved in any significant (*p* ≥ 0.14) interactions and was removed from the final model. Additionally, DMI was not significant (*p* = 0.74) and was also removed from the final model reported in Table 4. Like with Equation (4), both sides of the equation were multiplied by BW yielding:

UN (g/d) = [−0.253 + 0.00932 × MUN (mg/dL) + 0.0260 × CP (%, DM)] × BW (kg)(7)

### 3.3. Model Evaluation

**Figure 2:**
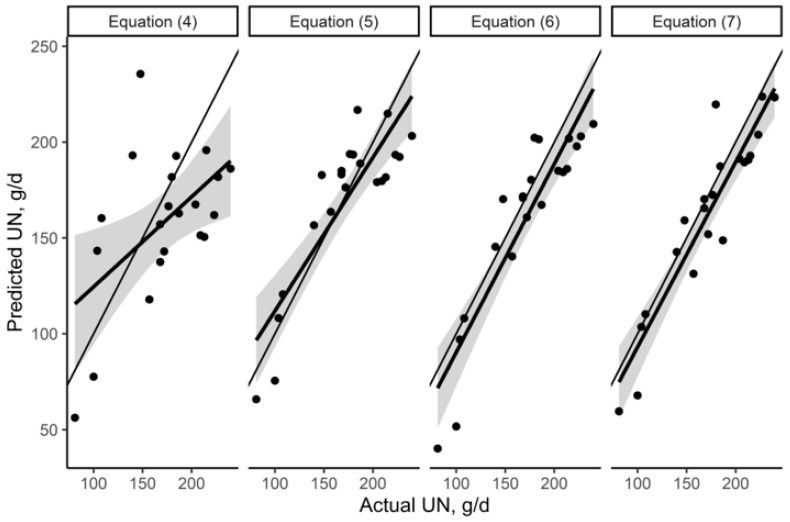
Predicted urinary nitrogen (UN) excretion (g/d) compared with actual UN excretion. Predicted values were calculated using newly developed equations: Equation (4): UN = 0.0214 × MUN × BW for total mixed ration (TMR) and UN = 0.0240 × MUN × BW for Pasture fed cows; Equation (5): UN = −209.4 + 6.8 × MUN + 12.4 × CP + 4.8 × DMI; Equation (6): UN = −281.3 + 6.7 × MUN + 13.2 × CP + 2.8 × DMI + 0.16 × BW; Equation (7): UN = (−0.253 + 0.00932 × MUN + 0.0260 × CP) × BW.

Table 5 displays the mean, standard deviation (SD), coefficient of variation (CV), and maximum and minimum values from the observed UN or predicted UN using Equations (4)–(7). Equation (4) on average underestimated UN by 13 g/d, and the predicted UN had a lower SD (5.5 less) than the observed values. Equations (6) and (7) also underestimated the average UN relative to the observed values by 10.9 g/d and 9.4 g/d, respectively. However, Equation (5) only underestimated UN by 2.3 g/d. This can be seen graphically in Figure 2.

Table 6 reports the comparative statistics between observed and predicted UN for Equations (4)–(7). Equation (4) had the worst performance compared with the other models. Equation (4) had the worst precision (r = 0.53; RMSEP = 42.4; RPE = 24.7) and only moderate agreement (CCC = 0.50). Including animal and dietary factors, as is the case for Equations (5)–(7), greatly improved model performance relative to MUN only (i.e., Equation (4)). This is seen where Equations (5)–(7) all had very good agreement (CCC ≥ 0.86) and relatively low RMSEP and RPE. Furthermore, by including BW as a predictor variable, there was improvement in model performance, where CCC, RMSEP, and RPE were all improved in Equation (6) relative to Equation (5). Finally, the approach used for Equation (7), where UN/BW was the dependent variable, not only removed the significance of a predictor variable (DMI), but also slightly improved model performance relative to Equation (6).

## 4. Discussion

We hypothesized that existing models developed from cows fed TMR diets would have significant biases when used to predict UN from cows fed fresh forages. The results of this current research support this hypothesis. This is seen by the significant linear prediction biases for Equations (1) and (3) and the large RMSEP and RPE for Equation (2). Furthermore, we set out to develop robust UN prediction equations that were applicable to TMR and pastoral dairy production systems. Based on the validation of the newly generated Equations (5)–(7), we have successfully achieved this goal. Below we discuss key findings that support these claims.

### 4.1. Performance of Established Equations

Equations (1) and (2) were inadequate for TMR-fed cows. Equation (1) had a large mean prediction bias, meaning that, on average Equation (1) overpredicted UN excretion by 31.4 g/d or 16% greater than the observed mean. When comparing prediction model estimates to the plasma urea content, Pacheco et al. [33] also noted that Equation (1) underpredicted UN excretion relative to an equation that predicts daily UN excretion based on BW, urinary creatinine, and urine N concentrations. Sannes et al. [34] also reported that Equation (1) underpredicted UN excretion, by 54.6 g/d (22% lower) in their instance. Equation (2) had a significant mean and linear bias for TMR-fed cows. Equation (2) underpredicted UN excretion by −24.7 g UN/d on average, or by 12.5% relative to the observed mean. When using a similar equation to Kauffman and St-Pierre [14] (see Kohn et al. [15]), UN excretion was overpredicted (32.7 g/d; 13% greater) for TMR-fed cows to a similar degree as in the current study [34]. Equation (1) appears to consistently underpredict and Equation (2) appears to consistently overpredict UN excretion. The reason for why Equation (1) underpredicts UN excretion may be due to the equation not accounting for animal BW or dietary conditions, like CP content. Why Equation (2) appears to consistently overpredict UN excretion is less clear. However, it may be related to a limited number of animal observations, resulting from one study, used for developing the equation (data collected from only 8 animals). Intuitively, developing an equation using an empirical dataset (as was the case by Kauffman and St-Pierre [14]) provides accurate equations for similar conditions, but does not guarantee accuracy for other conditions. Finally, Equations (1) and (2) had large RMSEP and RPE, indicating that these equations lacked precision. For these reasons, Equations (1) and (2) should not be used for TMR-fed cows as they provide inaccurate and imprecise estimates. 

Equation (3) performed well for TMR-fed cows. For the data from TMR-fed cows, there was no significant linear or mean prediction bias. There were also low RMSEP values relative to the other equations. Putting this into biological perspective for TMR-fed cows, the RMSEP was only 15.9% of the observed mean. These results make sense as the dataset used by Spek et al. [16] to develop Equation (3) included 55 out of the 174 treatment mean observations used in the current analysis. These results suggest that using Equation (3) is appropriate for TMR-fed cows.

Unlike for TMR-fed cows, none of the equations had adequate performance for fresh forage-fed cows. The prediction error was not consistent across the whole range of data for fresh forage-fed cows for Equations (1) and (3), as seen by the significant linear prediction biases. Furthermore, Equations (1) and (2) had large RMSEP, which were 25.7% and 33.5% of the observed mean, respectively. None of the equations had significant mean prediction biases for fresh forage-fed cows; however, due to the significant linear prediction biases of Equations (1) and (3) and the large RMSEP (indicating poor precision) seen for Equations (1) and (2), none of the equations are suitable for pastoral-based dairy systems.

When using residuals (observed UN minus predicted UN) as the dependent variable, all of the established UN prediction equations that we evaluated had a significant interaction between mean centered predicted values and diet type. This suggests that the models evaluated are not adequately accounting for some interfering variables that are associated with feeding TMR or fresh forages. There are intrinsic differences that exist between TMR-based and fresh forage-based systems (Table 1). Across the training and test data sets, cows fed fresh forage were offered 9% and 33% greater CP and NDF in the diet, respectively, than that offered to TMR-fed cows. The cows from the TMR experiments had 30%, 34%, and 23% greater BW, DMI, and N intake, respectively, than cows from fresh forage-based experiments did. Additionally, TMR-fed cows had 69% greater milk yield. However, fresh forage-fed cows had 27% greater MUN content than TMR-fed cows did. These observations make intuitive sense when considering the types of animals used in these production systems. Appuhamy et al. [35] reported that cows from experiments conducted in Australia and New Zealand (as an example of fresh forage-based systems) were fed diets composed of 88% forage, which contained greater content of CP and NDF than cows from North America (as an example of TMR-based systems). Moreover, in the Appuhamy et al. [35] dataset, North American cows had greater BW and daily milk yield than the Australian and New Zealand cows. The work by Appuhamy et al. [35] and the current data set highlights how cow type and phenotypical traits can differ based on the intensive nature of a production system. More extensive, grazing-based systems, such as those seen in New Zealand and Australia, are better suited to lighter cows with lower intakes, whereas intensive, TMR-based systems can utilize larger cows with greater levels of intake and performance. Therefore, the differences observed in the current dataset between the TMR-based and forage-based systems are expected and consistent with previous literature.

To assess how these intrinsic differences between intensive TMR-based and forage-based systems may explain why Equations (1)–(3) had such contrasting responses between the two systems, we regressed MUN, dietary CP, DMI, and BW against each equation’s residuals (observed UN minus predicted UN). Interestingly, even though all three equations included MUN as a predictor variable, as MUN increased, the residuals of all three equations decreased. This suggests that the regression coefficients for MUN in these equations are not adequate for the observations in the current experiment. Likewise, even though Equation (3) included CP content, as CP content increased, the residuals of Equation (3) decreased, again suggesting that the coefficient of CP content for Equation (3) is not adequate for the current dataset. The next interesting finding from this analysis is around DMI and BW. Dry matter intake had a positive linear relationship with Equation (1) residuals only and was not related to the other equations’ residuals. This makes sense for Equation (3), which included DMI as a predictor variable. The lack of relationship between DMI and Equation (2) residuals is likely because it includes BW as a model term and BW is known to have a strong positive relationship with DMI. In terms of BW relationship with equation residuals, it was not significant for Equation (2), which makes sense because this model includes BW as a predictor variable. However, it was significant for Equations (1) and (3), both of which did not include BW as a predictor variable. Kauffman and St-Pierre [14] in their development of Equation (2) demonstrated that the UN-to-MUN ratio was strongly influenced by BW. In fact, even Spek et al. [16] determined that BW was a significant factor in prediction UN; however, it was not included in the final model because it did not improve model fit in their instance. Ultimately, whenever a variable is significantly related to the equation residuals and it is included in the equation already (as seen with MUN for Equations (1)–(3) and CP for Equation (3)), it would suggest that the coefficients are not fully capturing the influence of the respective variable on UN excretion. Moreover, when a variable that is not included in the equation as a predictor variable is related to its residuals, as is seen with CP for Equation (1) and BW for Equations (1) and (3), this would suggest that the influence of this variable on UN is not being accounted for by the model. These results support that the divergence between TMR-fed and pasture-fed cows biases for Equations (1)–(3) is related to intrinsic biological aspects that differ between the two systems.

Further nutritive values exist that could influence the relationship of UN to MUN and subsequently explain the differences observed between TMR-fed and fresh forage-fed cows. However, the current analysis is limited to common values reported in all experiments composing the data frame. The water-soluble carbohydrate (WSC) and non-fiber carbohydrate content of diets are potential dietary characteristic that could influence the UN to MUN ratios. For example, increasing the WSC-to-CP ratio of the diet has resulted in reduced ruminal NH_3_ production both in vitro [36] and in vivo [37]. The reduction in ruminal NH_3_ production occurs by increasing the amount of N that is used for microbial cell protein production [38]. Ruminal NH_3_ that is not incorporated into microbial cell protein is absorbed from the rumen, and then, the NH_3_ is subsequently converted to urea in the liver, thereby contributing to UN excretion [39]. Furthermore, cows had reduced UN excretion and greater N use efficiency when they were fed a diet containing greater energy from concentrates (higher in starch) rather than from roughages [3]. Additional research is required to determine if predictive capabilities for UN excretion can be improved when additional dietary characteristics, such as water-soluble carbohydrate, non-fiber carbohydrate, starch, and fat content, are included in predictive equations. Exploring the ability of these dietary characteristics was not possible in the current study because we were limited to common values reported by all manuscripts in the data set.

### 4.2. Newly Developed Equations

There was a significant interaction between MUN and diet type seen in Equation (4). Equation (4) is similar to the approach taken by Kauffman and St-Pierre [14] when developing Equation (1). Kauffman and St-Pierre [14] stated that the coefficient of their UN prediction equation (0.0259) suggests a renal clearance rate (volume of blood flow through the kidneys) of 2.59 L/kg of BW per d. In comparison, Jonker et al. [13] reported a renal clearance rate of 2.10 L/kg of BW per d. Using this same logic as Kauffman and St-Pierre [14], based on Equation (4), this study would suggest a renal clearance rate of 2.14 L/kg of BW per d for TMR-fed cows and 2.40 L/kg of BW per d for fresh forage-fed cows. The renal clearance rate for TMR-fed cows is close to the estimate of Jonker et al. [13], which was also assessed with TMR-fed cows. However, it is not immediately obvious why fresh forage-fed cows would have a greater renal clearance rate than TMR-fed cows. However, when CP content and DMI were included in the models, the interaction between MUN and diet type disappeared. This may suggest that the greater renal clearance rate observed for fresh forage-fed cows may be related to dietary CP content and DMI, which is supported by the 16.8% greater CP content of the diets composing the training dataset. The greater CP content leading to faster renal clearance rates makes biological sense as well. As dietary CP content increases, the proportion of UN as urinary urea N increases [11]. Urea is an important osmolyte, meaning that greater urea content in urine would increase the renal clearance rate and subsequent urea excretion whenever the animals are not dehydrated [4]. 

Including MUN, DMI, and CP in Equation (5) removed the involvement of diet type in any interactions previously seen in Equation (4). This suggests that the differences in DMI and CP between pastoral and TMR dairy systems explain the variability that was previously accounted for by including the diet type × MUN interaction in Equation (4). This trend remained consistent for the other established equations, where diet type was not involved in a significant interaction with model terms for Equation (6), where MUN, CP, DMI, and BW were included as model terms, or Equation (7) where MUN, CP, and BW were included as model terms. One interesting observation made when developing the new Equations (6) and (7) was that MUN, CP, DMI, and BW were all significant terms when UN (g/d) was used as the dependent variable; however, when UN (g/d) per BW (kg) was used as the dependent variable when developing Equation (7), DMI was no longer a significant term in the model. While the actual cause for this is unclear, it does indicate that including BW in Equation (7) by this means (i.e., UN (g/d) per BW (kg) as the dependent variable) allowed it to explain a larger proportion of the variation and account for variability previously explained by DMI when developing Equation (6).

### 4.3. Performance of New Equations

Equation (4) performed the worst relative to the other developed models, with only moderate agreement (CCC = 0.50) and the worst predictive capabilities (RMSEP = 42.4 and RPE = 24.7%). This poor performance may be related to the data included in the test set. As discussed above, we speculated that the 16.8% greater MUN coefficient for fresh forage-fed cows compared with TMR-fed cows was related to the differences in dietary CP content between these two systems that existed for the training test set. However, this relationship did not exist in the test set; in fact, the CP content of the fresh forage diet was 7.7% less than the CP content of the TMR diets in the test set. Accordingly, we do not suggest using Equation (4) for TMR-fed or fresh forage-fed cows.

When going from Equation (4) (MUN only) to Equation (5) (MUN, CP, and DMI), the model performance was greatly improved. This was likewise observed by Spek et al. [16] when developing Equation (3). Furthermore, when BW was added as a predictor variable, as for Equations (6) and (7), it further improved the agreement between observed and predicted UN. Body weight was a significant factor for the analysis of Spek et al. [16] as well; however, they did not include it in the final models because it did not reduce RMSEP in their instance. Including BW in Equation (6) did not reduce RMSEP by an appreciable amount relative to Equation (5) in our instance either—only reducing RMSEP by 1.3%. Yet, we did observe that including BW slightly increased Pearson’s correlation and Lin’s CCC when comparing Equations (5) and (6). As mentioned previously, when UN (g/d) per BW (kg) was used as the dependent variable when developing Equation (7), DMI was not significant and only CP, MUN, and BW were used to predict UN. This also resulted in the equation with the greatest agreement (CCC = 0.91) and predictive capabilities (RMSEP = 19.5; RPE = 11.3%) relative to the other newly developed models. Furthermore, Equation (7) had an 11.8% and 13% lower RMSEP relative to Equations (5) and (6), respectively. Based on these results, we suggest that Equation (7) can be used to predict UN excretion from both TMR-based and pastoral-based dairy systems.

## 5. Conclusions

There is a need to accurately predict UN excretions of dairy cows using MUN due to the difficulty of measuring UN directly and the ease of monitoring MUN in dairy systems. Providing accurate and precise equations to predict UN from MUN is especially needed because policy makers are interested in monitoring MUN to inform policymaking decisions that limit nitrogen emissions from dairy farms [18] and producers need tools to make informed management decisions. This research highlighted that previously established UN prediction equations (Equations (1)–(3)) for dairy cows that were developed using data predominantly from TMR-fed cows are inappropriate for dairy cows fed predominantly fresh forage diets. Finally, newly developed equations based on MUN, dietary CP content, and BW provided estimates with better agreement than previously published equations when evaluated against observed UN excretion values for both TMR-fed and fresh forage-fed cows. These results suggest that the newly developed Equation (7) can be used to predict UN from dairy cows in TMR-based and pastoral-based systems.

## Figures and Tables

**Table 1:** Description of the data set generated from the literature used for model development (training set) and testing models (test set) where cows were fed either a total mixed ration (TMR) or fresh forage.

	Complete Data Set		Training Set		Test Set	
Items ^1^	TMR	Pasture	TMR	Pasture	TMR	Pasture
No. of experiments	41	10	38	7	3	3
No. of treatment obs.	143	31	132	21	11	10
Dietary Factors, Mean (SD)						
CP, % DM	16.7 (1.79)	18.2 (3.58)	16.7 (1.85)	19.5 (3.40)	16.8 (0.65)	15.5 (2.25)
NDF, % DM	32.6 (1.79)	43.5 (3.58)	33.1 (5.80)	44.4 (6.74)	27.7 (8.32)	41.7 (7.70)
Roughage, %	54.1 (9.09)	92.3 (13.24)	53.9 (9.30)	89.4 (15.18)	57.05 (3.26)	98.3 (3.54)
Animal Factors, Mean (SD)						
UN, g/d	197.0 (53.56)	195.1 (58.92)	196.7 (55.34)	221.0 (46.98)	199.7 (24.78)	140.8 (42.65)
MUN, mg/dL	13.1 (3.09)	16.7 (4.07)	13.2 (3.17)	18.5 (2.46)	12.2 (1.77)	12.8 (4.17)
BW, kg	647.6 (64.94)	497.3 (60.57)	647.5 (64.74)	508.6 (63.60)	648.7 (70.59)	473.6 (48.16)
DMI, kg/d	22.2 (2.59)	16.6 (2.85)	22.1 (2.65)	17.0 (3.25)	22.6 (1.82)	15.9 (1.65)
N intake, g/d	594.6 (99.89)	482.9 (108.93)	593.7 (103.21)	518.3 (84.67)	606.5 (44.21)	408.3 (120.47)
MY, kg/d	34.2 (18.14)	20.2 (5.41)	34.4 (18.84)	20.3 (6.46)	32.0 (4.50)	20.1 (2.18)
DIM, d	123.3 (69.42)	111.1 (55.22)	125.7 (70.16)	115.3 (57.73)	95.5 (55.55)	102.4 (51.32)

^1^ No. = number; obs = observation; UN = urinary nitrogen; MUN = milk urea nitrogen; BW = body weight; DMI = dry matter intake; N intake = nitrogen intake; MY = Milk Yield; DIM = days in milk.

**Table 2:** Performance of established urinary nitrogen (UN, g/d) prediction equations. Biases were determined by regressing the Observed minus Predicted values by the mean centered predicted estimates, using the experiment as a random effect and weighting for UN standard error of the mean (SEm).

Items ^1,2^	TMR ^3^	Pasture	*p*-Value ^4^
Equation (1) (Jonker et al., 1998)			
Mean Bias	31.4	−3.5	0.02
SEm	6.25	12.8	—
*p*-value	<0.01	0.78	—
Linear Bias	−0.003	−0.17	0.02
SEm	0.058	0.04	—
*p*-value	0.92	<0.01	—
RMSEP, g UN/d	51.8	50.2	—
RPE, %	26.3	25.7	—
Equation (2) (Kauffman and St-Pierre, 2001)			
Mean Bias	−24.7	−18.6	0.68
SEm	6.52	13.28	—
*p*-value	<0.01	0.17	—
Linear Bias	−0.3	−0.07	<0.01
SEm	0.041	0.046	—
*p*-value	<0.01	0.15	—
RMSEP	49.0	65.4	—
RPE, %	24.9	33.5	—
Equation (3) (Spek et al., 2013)			
Mean Bias	0.8	−15.2	0.10
SEm	4.19	8.59	—
*p*-value	0.86	0.08	—
Linear Bias	0.05	−0.15	<0.01
SEm	0.045	0.033	—
*p*-value	0.23	<0.01	—
RMSEP	31.3	32.8	—
RPE, %	15.9	16.8	—

^1^ RMSE = Root Mean Square Error; RPE = Relative Prediction Error, RMSE as a percent of mean observed UN. ^2^ *p*-value listed under Mean Bias and Linear bias tests if the biases are different from zero. ^3^ TMR = Total mixed ration. ^4^ *p*-value listed in this column tests if the Mean Bias or Linear bias differs between diet types.

**Table 3:** Regression equations relating milk urea nitrogen (MUN; mg/dL), dietary crude protein content (CP; %), dry matter intake (DMI; kg), or body weight (BW; kg) to observed urinary nitrogen (UN; g/d) minus predicted UN for three established equations. A significant slope indicates that the original equation did not adequately explain variation that could be accounted for by the regressor of the current analysis.

Variables ^1^	Regression Equation ^2^
Milk Urea Nitrogen, mg/dL (MUN)	
Observed—Predicted by Equation (1)	–1.5 (0.43) ** × MUN + 45.1 (8.41) **
Observed—Predicted by Equation (2)	–1.8 (0.43) ** × MUN + 0.54 (8.72)
Observed—Predicted by Equation (3)	–1.2 (0.35) ** × MUN + 13.8 (6.13) *
Crude Protein, % (CP)	
Observed—Predicted by Equation (1)	0.3 (0.83) × CP + 17.9 (15.5)
Observed—Predicted by Equation (2)	0.5 (0.85) × CP − 33.9 (15.94) *
Observed—Predicted by Equation (3)	–1.7 (0.65) ** × CP + 26.3 (11.79) *
Dry Matter Intake, kg (DMI)	
Observed—Predicted by Equation (1)	5.6 (0.84) ** × DMI − 94.4 (18.6) **
Observed—Predicted by Equation (2)	1.3 (0.97) × DMI − 52.5 (21.4) *
Observed—Predicted by Equation (3)	0.9 (0.71) × DMI − 22.7 (15.4)
Body Weight, kg (BW)	
Observed—Predicted by Equation (1)	0.2 (0.045) ** × BW − 122.7 (28.74) **
Observed—Predicted by Equation (2)	–0.08 (0.051) × BW + 23.1 (32.24)
Observed—Predicted by Equation (3)	0.1 (0.033) ** × BW − 69.07 (20.9)

^1^ Equation (1): UN = 12.54 × MUN [13]; Equation (2): UN = 0.0259 × MUN × BW [14]; Equation (3): UN = –148.8 + 8.06 × MUN + 8.91 × CP + 4.06 × DMI [16]. ^2^ values with parenthesis are the standard error of the mean. * *p* < 0.05; ** *p* < 0.01.

**Table 4:** Developed equations to predict urinary nitrogen (g/d; UN) or UN per kg of body weight (kg; BW) as a function of milk urea nitrogen (MUN; mg/dL) only, dietary crude protein (%; CP), dry matter intake (kg; DMI), or BW from cows fed a total mixed ration (TMR) or fresh forage diet (Pasture).

		Random Effect Variance, % ^1^	
Equations ^3^	RSD ^2^	Study	Residual
For TMR: UN/BW = 0.0214 (0.00156) × MUN For Pasture: UN/BW = 0.0240 (0.000704) × MUN	0.02	92.4	7.6
UN = −209.4 (24.40) + 6.8 (0.80) × MUN + 12.4 (1.51) × CP + 4.8 (0.74) × DMI	11.8	79.8	20.2
UN = −281.3 (31.04) + 6.7 (0.77) × MUN + 13.2 (1.47) × CP + 2.8 (0.93) × DMI + 0.16 (0.046) × BW	11.4	79.2	20.8
UN/BW = −0.253 (0.0323) + 0.00932 (0.00132) × MUN + 0.0260 (0.00250) × CP	0.019	82.9	17.1

^1^ Random Effect Variance, % = the proportion of residual variance accounted for by the random effect (study) or unaccounted for (residual). ^2^ RSD = residual standard deviation. ^3^ Values within parenthesis are the standard error of the parameter.

**Table 5:** Descriptive statistics of the test data set for observed urinary nitrogen excretion (UN; g/d) and UN predicted from models.

		Model Predicted ^1^			
Items ^2^	Observed ^3^	Equation (4)	Equation (5)	Equation (6)	Equation (7)
Mean	171.6	158.6	169.3	160.7	162.2
SD	45.1	39.6	41.8	48.5	46.9
CV	26.3	25.1	24.7	30.2	28.9
Max. Value	239.8	235.6	216.8	209.4	223.8
Min. Value	81.0	56.2	65.8	40.2	59.5

^1^ Equation (4): UN = 0.0214 × milk urea nitrogen (MUN, mg/dL) × body weight (BW) for TMR and UN = 0.0240 × MUN × BW for Pasture fed cows; Equation (5): UN = −209.4 + 6.8 × MUN + 12.4 × crude protein (CP, %) + 4.8 × DMI; Equation (6): UN = −281.3 + 6.7 × MUN + 13.2 × CP + 2.8 × DMI + 0.16 × BW; Equation (7): UN = (−0.253 + 0.00932 × MUN + 0.0260 × CP) × BW. ^2^ Mean, g UN/d; SD = standard deviation, g UN/d; CV (coefficient of variation), % = 100 × SD/Mean; Max. = Maximum, g UN/d; Min. = minimum, g UN/d. ^3^ Actual = reported means from test set.

**Table 6:** Statistics used to compare the ability of newly developed and established models to predict UN based on a test set of data acquired from the literature.

	Comparative Statistics ^1^						
Comparisons ^2^	r	*u*	*v*	C_b_	CCC	RMSEP	RPE
Observed vs. Equation (4)	0.53	−0.33	0.88	0.94	0.50	42.4	24.7
Observed vs. Equation (5)	0.86	−0.06	0.93	0.99	0.86	22.4	13.1
Observed vs. Equation (6)	0.91	−0.24	1.08	0.97	0.89	22.1	12.8
Observed vs. Equation (7)	0.93	−0.21	1.04	0.98	0.91	19.5	11.3

^1^ r = Pearson’s Correlation; *u* = location shift, the mean bias; *v* = scale shift, a measure of the difference in SD; C_b_ = bias correction factor; CCC = Lin’s Concordance Correlation Coefficient, calculated as C_b_ × r [29,30]; RMSEP = Root Means Square Error of Prediction, g UN/d; RPE = relative prediction error, calculated as RMSEP as a percent of the observed UN mean (171.6). ^2^ Equation (4): UN = 0.0214 × MUN × BW for TMR and UN = 0.0240 × MUN × BW for Pasture fed cows; Equation (5): UN = −209.4 + 6.8 × MUN + 12.4 × CP + 4.8 × DMI; Equation (6): UN = −281.3 + 6.7 × MUN + 13.2 × CP + 2.8 × DMI + 0.16 × BW; Equation (7): UN = (−0.253 + 0.00932 × MUN + 0.0260 × CP) × BW.

## Data Availability

Data used to perform analysis are available as Supplementary Materials.

## Supplementary Materials

The following supporting information can be downloaded at: https://www.mdpi.com/article/10.3390/ani13040620/s1, Table S1: PRISMA 2020 Checklist; Table S2: Compiled data of 51 experiments used to develop and compare predictive equations for urinary nitrogen excretion. Complete reference list of experiments composing the data frame. R code used for all statistical analysis and figure creation.
