# Supplementary material for: Meta-Regression to Develop Predictive Equations for Urinary Nitrogen Excretion of Lactating Dairy Cows
Source: Animals (Basel). 2023 Feb 10;13(4):620. doi: 10.3390/ani13040620 (PMC9951755; doi:10.3390/ani13040620)
Supplement: Supplementary file 1 [file animals-13-00620-s001.zip › animals-2177691-supplementary.pdf]

Table S1. PRISMA 2020 Checklist.

PRISMA 2020 Checklist

Section and Topic	Item #	Checklist item	Location where item is reported
**TITLE**			
Title	1	Identify the report as a systematic review.	1
**ABSTRACT**			
Abstract	2	See the PRISMA 2020 for Abstracts checklist.	1
**INTRODUCTION**			
Rationale	3	Describe the rationale for the review in the context of existing knowledge.	2-3
Objectives	4	Provide an explicit statement of the objective(s) or question(s) the review addresses.	3
**METHODS**			
Eligibility criteria	5	Specify the inclusion and exclusion criteria for the review and how studies were grouped for the syntheses.	3-4
Information sources	6	Specify all databases, registers, websites, organisations, reference lists and other sources searched or consulted to identify studies. Specify the date when each source was last searched or consulted.	3-4
Search strategy	7	Present the full search strategies for all databases, registers and websites, including any filters and limits used.	3-4
Selection process	8	Specify the methods used to decide whether a study met the inclusion criteria of the review, including how many reviewers screened each record and each report retrieved, whether they worked independently, and if applicable, details of automation tools used in the process.	3-4
Data collection process	9	Specify the methods used to collect data from reports, including how many reviewers collected data from each report, whether they worked independently, any processes for obtaining or confirming data from study investigators, and if applicable, details of automation tools used in the process.	4
Data items	10a	List and define all outcomes for which data were sought. Specify whether all results that were compatible with each outcome domain in each study were sought (e.g. for all measures, time points, analyses), and if not, the methods used to decide which results to collect.	3-4
	10b	List and define all other variables for which data were sought (e.g. participant and intervention characteristics, funding sources). Describe any assumptions made about any missing or unclear information.	3-4
Study risk of bias assessment	11	Specify the methods used to assess risk of bias in the included studies, including details of the tool(s) used, how many reviewers assessed each study and whether they worked independently, and if applicable, details of automation tools used in the process.	4
Effect measures	12	Specify for each outcome the effect measure(s) (e.g. risk ratio, mean difference) used in the synthesis or presentation of results.	Table 1

Synthesis methods	13a	Describe the processes used to decide which studies were eligible for each synthesis (e.g. tabulating the study intervention characteristics and comparing against the planned groups for each synthesis (item #5)).	3-4
	13b	Describe any methods required to prepare the data for presentation or synthesis, such as handling of missing summary statistics, or data conversions.	3-4
	13c	Describe any methods used to tabulate or visually display results of individual studies and syntheses.	4
	13d	Describe any methods used to synthesize results and provide a rationale for the choice(s). If meta-analysis was performed, describe the model(s), method(s) to identify the presence and extent of statistical heterogeneity, and software package(s) used.	6-7
	13e	Describe any methods used to explore possible causes of heterogeneity among study results (e.g. subgroup analysis, meta-regression).	6
	13f	Describe any sensitivity analyses conducted to assess robustness of the synthesized results.	6
Reporting bias assessment	14	Describe any methods used to assess risk of bias due to missing results in a synthesis (arising from reporting biases).	No missing results in data set
Certainty assessment	15	Describe any methods used to assess certainty (or confidence) in the body of evidence for an outcome.	6-7

PRISMA 2020 Checklist

Section and Topic	Item #	Checklist item	Location where item is reported
**RESULTS**			
Study selection	16a	Describe the results of the search and selection process, from the number of records identified in the search to the number of studies included in the review, ideally using a flow diagram.	Figure 1
	16b	Cite studies that might appear to meet the inclusion criteria, but which were excluded, and explain why they were excluded.	3-4
Study characteristics	17	Cite each included study and present its characteristics.	Included as Supplemental Materials
Risk of bias in studies	18	Present assessments of risk of bias for each included study.	7-8
Results of individual studies	19	For all outcomes, present, for each study: (a) summary statistics for each group (where appropriate) and (b) an effect estimate and its precision (e.g. confidence/credible interval), ideally using structured tables or plots.	Supplemental Table 1
	20a	For each synthesis, briefly summarise the characteristics and risk of bias among contributing studies.	7-8

Results of syntheses	20b	Present results of all statistical syntheses conducted. If meta-analysis was done, present for each the summary estimate and its precision (e.g.<br>confidence/credible interval) and measures of statistical heterogeneity. If comparing groups, describe the direction of the effect.	Table 2-6
	20c	Present results of all investigations of possible causes of heterogeneity among study results.	Table 3
	20d	Present results of all sensitivity analyses conducted to assess the robustness of the synthesized results.	Table 6
Reporting biases	21	Present assessments of risk of bias due to missing results (arising from reporting biases) for each synthesis assessed.	No missing results
Certainty of evidence	22	Present assessments of certainty (or confidence) in the body of evidence for each outcome assessed.	Table 6
**DISCUSSION**			
Discussion	23a	Provide a general interpretation of the results in the context of other evidence.	12
	23b	Discuss any limitations of the evidence included in the review.	14-15
	23c	Discuss any limitations of the review processes used.	14-15
	23d	Discuss implications of the results for practice, policy, and future research.	12-16
**OTHER INFORMATION**			
Registration and protocol	24a	Provide registration information for the review, including register name and registration number, or state that the review was not registered.	Not registered
	24b	Indicate where the review protocol can be accessed, or state that a protocol was not prepared.	Not prepared
	24c	Describe and explain any amendments to information provided at registration or in the protocol.	N/A
Support	25	Describe sources of financial or non-financial support for the review, and the role of the funders or sponsors in the review.	16
Competing interests	26	Declare any competing interests of review authors.	16
Availability of data, code and other materials	27	Report which of the following are publicly available and where they can be found: template data collection forms; data extracted from included studies; data used for all analyses; analytic code; any other materials used in the review.	Included as Supplemental Materials

From: Page MJ, McKenzie JE, Bossuyt PM, Boutron I, Hoffmann TC, Mulrow CD, et al. The PRISMA 2020 statement: an updated guideline for reporting systematic reviews. BMJ 2021;372:n71. doi: 10.1136/bmj.n71

For more information, visit: http://www.prisma-statement.org/

Table S2. Compiled data of 51 experiments used to develop and compare predictive equations for urinary nitrogen excretion.

Citation	Training/ Test set	Exp. Design[1]	Breed[1]	Diet[1]	n	Diet CP[1]	Diet NDF[1]	DMI[1]	BW[1]	MUN[1]	UN[1]	UN/BW SEm
[1]	Training	LS	H	TMR	6	12.9	31.3	23.7	646	10.3	115	0.012
	Training	LS	H	TMR	6	13.4	30.6	23.6	646	10.8	133	0.012
	Training	LS	H	TMR	6	15.4	31.6	25.2	646	14.4	188	0.012
[2]	Training	LS	H	TMR	6	16.4	26.3	24.8	660	16.2	227	0.015
	Training	LS	H	TMR	6	16.5	32.4	24.8	660	16.8	233	0.015
[3]	Training	LS	H	TMR	16	15.2	29.5	24.1	669	11.8	168	0.011
	Training	LS	H	TMR	4	15.9	29.2	22.1	669	12.9	179	0.011
	Training	LS	H	TMR	4	15.8	30.1	23.7	669	13.9	201	0.011
	Training	LS	H	TMR	4	15.8	29.7	23.8	669	13.6	207	0.011
	Training	LS	H	TMR	4	15.7	30.3	23.3	669	14	213	0.011
	Training	LS	H	TMR	16	16.5	30.1	22.3	669	15.4	232	0.011
[4]	Training	LS	H	TMR	8	15.4	27.3	24	637	14.3	197	0.02
	Training	LS	H	TMR	8	16.5	27.6	24.7	640	14.9	229	0.02
	Training	LS	H	TMR	8	17.2	27.9	25	639	15.2	241	0.02
	Training	LS	H	TMR	8	17.4	28.1	24.8	639	15.4	248	0.02
[5]	Training	CRD	H	TMR	3	18.4	26.2	21.6	773	13.6	235	0.019
	Training	CRD	H	TMR	3	18	25.8	21.8	773	13.2	236	0.019
	Training	CRD	H	TMR	3	18	26.2	22.4	773	13.5	242	0.019
	Training	CRD	H	TMR	3	18.2	26	22.2	773	14.1	249	0.019
[6]	Training	LS	H	TMR	6	16.8	24.7	19.9	630	15.5	195	0.019
	Training	LS	H	TMR	6	17.3	28.6	20.6	628	15.5	198	0.019
	Training	LS	H	TMR	6	17.4	32.2	21.4	627	17.4	211	0.019
[7]	Training	LS	H	TMR	12	16.2	32.3	23.4	705	11.1	204	0.012
	Training	LS	H	TMR	12	16.4	33.8	24.4	707.5	10	209	0.012
	Training	LS	H	TMR	12	16.5	36.3	24.8	710.5	9.9	213	0.012
	Training	LS	H	TMR	12	16.9	37.8	25.2	714	10.6	223	0.012
[8]	Test	LS	H	TMR	9	16.3	35.4	24.9	728	14	222	0.011
	Test	LS	H	TMR	9	16.1	32.4	26.2	734	12.7	224	0.011
	Test	LS	H	TMR	9	16.9	37.8	21.9	732	15.8	228	0.011
[9]	Training	LS	H	TMR	12	16.3	35.6	23.3	712.5	6.36	157	0.011
	Training	LS	H	TMR	12	16.5	36.2	23.8	714.7	10.51	174	0.011
	Training	LS	H	TMR	12	16.4	28.2	21.7	718.5	14.05	241	0.011
	Training	LS	H	TMR	12	16.6	29.6	23.8	710.4	14.93	256	0.011
[10]	Training	LS	H	TMR	32	17.9	31.3	23.4	703	10.6	215	0.008
	Training	LS	H	TMR	32	17.9	31.3	22.6	703	10.7	214	0.008
	Training	LS	H	TMR	32	17.9	31.3	23.8	703	10.4	215	0.008
	Training	LS	H	TMR	32	17.9	31.3	23.2	703	10.4	221	0.008

Table S2. continued

Citation	Training/ Test set	Exp. Design[1]	Breed[1]	Diet[1]	n	Diet CP[1]	Diet NDF[1]	DMI[1]	BW[1]	MUN[1]	UN[1]	UN/BW SEm
[11]	Training	CO	H	TMR	32	16.6	30.2	26.1	647	10.8	193	0.011
	Training	CO	H	TMR	32	16.6	30.2	26.0	647	10.5	199	0.011
[12]	Training	LS	H	TMR	32	16.5	33.7	24.9	682	11.1	204	0.006
	Training	LS	H	TMR	32	16.5	33.7	23.7	682	10.5	206	0.006
	Training	LS	H	TMR	32	16.5	33.7	24.8	682	10.5	211	0.006
	Training	LS	H	TMR	32	16.5	33.7	24.3	682	9.3	203	0.006
[13]	Training	LS	H	TMR	8	16.2	38.4	20.2	658	9.7	133	0.014
	Training	LS	H	TMR	8	19.9	40.2	21.3	658	15.7	252	0.014
	Training	LS	H	TMR	8	20.1	38.3	20.7	658	16.7	257	0.014
	Training	LS	H	TMR	8	19.7	36.1	22	658	17.1	261	0.014
[14]	Training	LS	H	TMR	28	16.3	24.1	23.7	644	14.4	188	0.008
	Training	LS	H	TMR	28	16.6	23.8	25.4	644	14.1	201	0.008
	Training	LS	H	TMR	28	17	23.5	26.5	644	13.9	215	0.008
	Training	LS	H	TMR	28	17.4	23.3	26.8	644	13.8	217	0.008
[15]	Training	LS	F	Pasture	8	13.2	45.7	15.3	418	5.6	81	0.006
	Training	LS	F	Pasture	8	13.2	45.7	14.3	418	7.73	100	0.006
	Training	LS	F	Pasture	8	17.6	46	15.6	418	11.75	157	0.006
	Training	LS	F	Pasture	8	17.6	46	14.5	418	16.23	187	0.006
[16]	Training	CRD	HF	Pasture	8	15.2	58.9	16	549	19.26	153.7	0.011
	Training	CRD	HF	Pasture	8	15.2	58.9	14.8	549	20.1	165	0.011
[17]	Training	LS	H	TMR	20	19.9	35.4	21.7	563	13.39	237	0.03
	Training	LS	H	TMR	23	20.6	38.9	20.6	563	17.48	289	0.03
	Training	LS	H	TMR	21	19.6	34.9	21.4	563	14.46	364	0.03
	Training	LS	H	TMR	22	20.6	40.4	20.1	563	16.96	396	0.03
[18]	Training	CRD	NS	TMR	8	17.3	38.2	17.8	639.3	14.3	187.5	0.02
	Training	CRD	NS	TMR	8	17.3	38.2	19.9	663.9	14.5	202.5	0.019
[19]	Training	LS	F	TMR	6	16	36.6	20	600	10.7	138	0.01
	Training	LS	F	TMR	6	17	35.8	20	600	11.9	157	0.01
	Training	LS	F	TMR	6	16.8	37	18.2	600	12.9	158	0.01
[20]	Training	LS	SR	TMR	14	16.8	35.2	19.1	607	13.669	187	0.011
	Training	LS	SR	TMR	14	17.7	35.2	20.1	607	16.86	237	0.011
	Training	LS	SR	TMR	14	17.7	35.1	20	607	16.33	244	0.011
[21]	Training	LS	H	TMR	4	18	25.6	18.6	487	12.4	173	0.023
	Training	LS	H	TMR	8	17	26	22.7	578	11.9	194	0.017
	Training	LS	H	TMR	8	17.7	25.4	21.1	575	12.6	202	0.017
	Training	LS	H	TMR	8	18.1	26.9	24.4	581	12.5	213	0.017
	Training	LS	H	TMR	8	18.9	23.9	23.6	577	13.4	223	0.017
	Training	LS	H	TMR	4	17.9	25.6	27.3	669	12.8	243	0.016

Table S2. continued

Citation	Training/ Test set	Exp. Design[1]	Breed[1]	Diet[1]	n	Diet CP[1]	Diet NDF[1]	DMI[1]	BW[1]	MUN[1]	UN[1]	UN/BW SEm
[22]	Training	LS	H	TMR	6	16.4	35.9	20.4	687	10.4	163	0.006
	Training	LS	H	TMR	6	17.3	38.5	21	687	13.8	186	0.006
	Training	LS	H	TMR	6	17.8	39.1	20.4	687	13.7	193	0.006
[23]	Training	CO	H	TMR	8	15.2	35.7	20.7	721	11.9	227	0.02
	Training	CO	H	TMR	8	16.3	34.6	21	721	13.3	272	0.02
[24]	Training	CRD	H	TMR	6	17.6	17.9	20.85	685	12.4	179.8	0.022
	Training	CRD	H	TMR	6	17.9	14	20	685	12.4	227.1	0.022
	Training	CRD	H	TMR	6	17.7	15.1	22.2	685	12.7	239.8	0.022
[25]	Training	LS	H	TMR	9	16.2	29.7	23	688	11.8	185	0.013
	Training	LS	H	TMR	9	15.7	28.6	22.8	688	13.4	188	0.013
	Training	LS	H	TMR	9	16.8	30.7	21.7	688	13.8	216	0.013
[26]	Test	CRD	HF x J	Pasture	5	18.4	43.1	19.1	520	11.01	168	0.021
	Test	CRD	HF x J	Pasture	5	18.4	43.1	18.7	520	15.69	215	0.021
[27]	Training	CO	H	TMR	4	19.7	39.6	23.1	768	22.1	301	0.008
	Training	CO	H	TMR	4	19.7	39.6	24.4	768	24.2	309	0.008
[28]	Training	CO	H	TMR	4	19.1	34.1	21.5	715	15.6	233	0.025
	Training	CO	H	TMR	4	19.5	26.1	24.2	715	18.2	279	0.025
[29]	Training	CO	H and H x SR	Pasture	12	16.4	43.2	20.7	579	18.9	193	0.019
	Training	CO	H x SR	Pasture	6	17	43.3	20.4	573	20.7	202	0.018
	Training	CO	H and H x SR	Pasture	12	17	43.3	21	582	20.9	208	0.019
	Training	CO	H	Pasture	6	16.8	43.3	21.5	583	20.7	220	0.018
	Training	CO	H and H x SR	Pasture	12	17.4	43.3	21	571	22.5	231	0.019
[30]	Training	CRD	BS and H	TMR	6	17.4	47.4	20.3	671	14.2	169	0.013
	Training	CRD	BS and H	TMR	6	15.7	50.7	17.2	679	11	103	0.013
	Training	CRD	BS and H	TMR	6	12.8	52.9	14	670	8.22	76	0.013
	Training	CRD	BS and H	TMR	6	14.5	45.4	19	663	11.5	100	0.013
[31]	Training	CO	J	TMR	12	18.9	43.4	16.7	480.1	20.1	215	0.024
	Training	CO	J	TMR	12	19	44.2	17.1	483	20.9	218.7	0.024
[32]	Training	LS	H and J	TMR	8	13.3	33.6	20.1	574	6.09	93	0.01
	Training	LS	H and J	TMR	8	14	40.9	20.5	574	6.83	101	0.01
	Training	WP/SP	J	TMR	4	15.2	37.1	16.9	470	9.47	118	0.013
	Training	WP/SP	H	TMR	4	15.3	37.1	24.4	678	9.44	166	0.009
	Training	LS	H and J	TMR	8	16.5	41.6	21.2	574	12.36	185	0.01
	Training	LS	H and J	TMR	8	17.2	32.4	20.8	574	12.55	190	0.01

Table S2. continued

Citation	Training/ Test set	Exp. Design[1]	Breed[1]	Diet[1]	n	Diet CP[1]	Diet NDF[1]	DMI[1]	BW[1]	MUN[1]	UN[1]	UN/BW SEm
[33]	Training	LS	NR	TMR	32	14.2	39.4	19.8	566	11.23	156.4	0.013
	Training	LS	NR	TMR	32	14.2	39.4	19	566	9.8	155.4	0.013
	Training	LS	NR	TMR	32	11.8	39.9	18.9	566	7.46	94.5	0.019
	Training	LS	NR	TMR	32	13.4	39.2	19.9	566	9.36	135.6	0.019
	Training	LS	NR	TMR	32	14.9	39.2	19.6	566	11.68	182	0.019
	Training	LS	NR	TMR	32	16.7	39.1	19.1	566	13.47	211.5	0.019
[34]	Training	CRD	H	TMR	9	16.4	30	20.6	556.1	13.2	167.9	0.015
	Training	CRD	H	TMR	9	15.9	28.5	22.5	561.4	11.9	172	0.015
	Training	CRD	H	TMR	9	16.7	30.6	20.9	551.9	14.1	176.4	0.015
	Training	CRD	H	TMR	9	16.7	28.5	23.5	573.9	15.7	184.3	0.014
[35]	Training	LS	A	TMR	4	12.7	—	19.8	668	5.93	103	0.012
	Training	LS	A	TMR	4	15.7	—	19.7	668	12.3	156	0.012
	Training	LS	A	TMR	4	16.2	—	19.7	668	12.4	169	0.012
	Training	LS	A	TMR	4	15.8	—	19.8	668	12.7	174	0.012
[36]	Training	CO	H	TMR	8	18.4	34.6	21.9	717	10.82	201	0.016
	Training	CO	H	TMR	8	18.3	34.6	22.6	717	12.31	240	0.016
[37]	Training	CO	F	Pasture	6	22.1	46.3	10.5	410.3	18.2	200	0.021
	Training	CO	F	Pasture	6	23	47.3	10.3	410.3	20.17	203	0.021
	Training	CO	F	Pasture	6	23	47.3	14	410.3	21	232	0.021
	Training	CO	F	Pasture	6	22.1	46.3	14.6	410.3	19.89	244	0.021
[38]	Test	CRD	HF x J	Pasture	4	13.4	27.3	14.9	506	11.8	103.76	0.037
	Test	CRD	HF x J	Pasture	4	13.4	27.3	15.5	506	13.2	108.1	0.037
	Test	CRD	HF x J	Pasture	4	14.9	46.5	15.3	506	15.9	139.85	0.037
	Test	CRD	HF x J	Pasture	4	14.9	46.5	15.8	506	19.4	147.83	0.037
[39]	Training	Incomplete Cross-over	HF	Pasture	8	18.8	34.1	17.4	483	13.8	197	0.018
	Training	Incomplete Cross-over	HF	Pasture	8	20	36.5	16.8	483	15.06	224	0.018
	Training	Incomplete Cross-over	HF	Pasture	8	23.1	41.3	14.8	483	16.85	263	0.018
	Training	Incomplete Cross-over	HF	Pasture	8	21.9	38.9	16.5	483	16.55	271	0.018
[40]	Training	LS	H	TMR	9	15.4	30.1	21.54	595	14.8	108.2	0.037
	Training	LS	H	TMR	9	15.4	32.4	21.21	595	16.3	134.1	0.037
	Training	LS	H	TMR	9	15.4	30.9	21.91	595	15	147.5	0.037
[41]	Training	LS	H	TMR	9	18.5	31.1	23.5	578	15.9	173.9	0.03
	Training	LS	H	TMR	9	18.5	31.8	24	578	16.6	184.3	0.03
	Training	LS	H	TMR	9	18.5	33.6	24.8	578	17.4	204.9	0.03

Table S2. continued

Citation	Training/ Test set	Exp. Design[1]	Breed[1]	Diet[1]	n	Diet CP[1]	Diet NDF[1]	DMI[1]	BW[1]	MUN[1]	UN[1]	UN/BW SEm
[42]	Training	CO	J	TMR	12	15.7	20.7	17.3	467	9.78	123	0.014
	Training	CO	J	TMR	12	16.2	29.8	17.6	467	9.7	150	0.014
[43]	Training	LS	H	TMR	8	14.9	36.3	25.5	711	12.3	152.8	0.023
	Training	LS	H	TMR	8	14.9	35.6	27	711	13	169.7	0.023
	Training	LS	H	TMR	8	17.4	38.6	27	711	16	248.5	0.023
	Training	LS	H	TMR	8	17.8	37	25.2	711	19.5	263.9	0.023
[44]	Test	LS	H	TMR	12	15.3	27.6	19.9	655	11.7	149	0.018
	Test	LS	H	TMR	12	16.9	24.4	20.2	656	13.8	181	0.018
	Test	LS	H	TMR	12	17.3	30.9	19.6	653	14.9	204	0.018
	Test	LS	H	TMR	12	18.9	27.8	19.9	654	17	237	0.018
[45]	Training	CRD	HF	Pasture	8	23.1	36.4	15.7	459	18.2	271	0.025
	Training	CRD	HF	Pasture	8	23.1	36.4	15.2	459	21	271	0.025
[46]	Training	CRD	H	TMR	8	9.4	35.9	16.8	614	4.8	39.5	0.017
	Training	CRD	H	TMR	8	11.1	35.6	19.9	614	6.3	51.6	0.017
	Training	CRD	H	TMR	8	14.1	35.2	20	614	14	139.4	0.017
[47]	Training	CRD	H	Pasture	4	14.2	51.8	19.9	560	14.09	141.6	0.027
	Training	CRD	H	Pasture	4	14.3	51.8	19.1	560	15.78	150.7	0.027
[48]	Test	LS	H	TMR	16	17	34.3	25.54	690	13.3	209.6	0.015
	Test	LS	H	TMR	16	17.4	34	25.73	690	14.3	233.9	0.015
	Test	LS	H	TMR	16	18.5	34.4	26.82	690	16.6	274	0.015
	Test	LS	H	TMR	16	19.6	34	26.27	690	18.7	289.3	0.015
[49]	Test	CO	F	Pasture	16	22.7	42.1	18.2	542	17.43	298.2	0.023
	Test	CO	F	Pasture	16	22.7	42.1	18.1	542	17.75	302.2	0.023
[50]	Training	LS	H	TMR	16	17.5	39.9	23.1	664	11.2	162	0.023
	Training	LS	H	TMR	16	17.3	29.5	23.1	669	11.2	112	0.023
	Training	LS	H	TMR	16	17.4	34.7	24.9	667	11.35	151	0.023
	Training	LS	H	TMR	16	17.3	26.6	24.1	671	11.3	167	0.023
[51]	Training	CRD	H	TMR	12	17.9	31.3	23.7	632	10.8	206	0.017
	Training	CRD	H	TMR	12	16.5	27.5	24.8	652	11.2	211	0.017
	Training	CRD	H	TMR	12	17.3	26.3	22.2	668	12.3	247	0.016
	Training	CRD	H	TMR	12	18	27.1	25.3	668	13.4	251	0.016

[1] Exp. Design = Experimental Design: LS = Latin Square, CRD = Completely Randomized Design, CO = Cross-over, WP/SP = Whole Plot-Split Plot; Breed: H = Holstein, J = Jersey, F = Friesian, HF = Holstein-Friesian, NS = not specified, SR = Swiss Red, HF x J = Holstein-Friesian Jersey cross, H x SR = Holstein x Swiss Red cross, A = Ayrshire; Diet: TMR = total mixed ration, Pasture = Fresh forage fed; Diet CP = dietary Crude Protein, % of DM; Diet NDF = dietary neutral detergent fiber, % of DM; DMI = dry matter intake, kg; BW = body weight, kg; MUN = milk urea nitrogen, mg/dL; UN = urine nitrogen excretion, g/d; UN/BW SEM = the UN (g/d) per BW (kg) standard error of the mean, this value was used to calculate the weighting factor used in the meta-regression analyses.

References

1. Agle, M.; Hristov, A.N.; Zaman, S.; Schneider, C.; Ndegwa, P.; Vaddella, V.K. The Effects of Ruminally Degraded Protein on Rumen Fermentation and Ammonia Losses from Manure in Dairy Cows. *J. Dairy Sci.* **2010**, *93*, 1625–1637, doi:10.3168/jds.2009-2579.
2. Agle, M.; Hristov, A.N.; Zaman, S.; Schneider, C.; Ndegwa, P.M.; Vaddella, V.K. Effect of Dietary Concentrate on Rumen Fermentation, Digestibility, and Nitrogen Losses in Dairy Cows. *J. Dairy Sci.* **2010**, *93*, 4211–4222, doi:10.3168/jds.2009-2977.
3. Aguerre, M.J.; Capozzolo, M.C.; Lencioni, P.; Cabral, C.; Wattiaux, M.A. Effect of Quebracho-Chestnut Tannin Extracts at 2 Dietary Crude Protein Levels on Performance, Rumen Fermentation, and Nitrogen Partitioning in Dairy Cows. *J. Dairy Sci.* **2016**, *99*, 4476–4486, doi:10.3168/jds.2015-10745.
4. Arndt, C.; Powell, J.M.; Aguerre, M.J.; Wattiaux, M.A. Performance, Digestion, Nitrogen Balance, and Emission of Manure Ammonia, Enteric Methane, and Carbon Dioxide in Lactating Cows Fed Diets with Varying Alfalfa Silage-to-Corn Silage Ratios. *J. Dairy Sci.* **2015**, *98*, 418–430, doi:10.3168/jds.2014-8298.
5. Barros, T.; Powell, J.M.; Danes, M.A.C.; Aguerre, M.J.; Wattiaux, M.A. Relative Partitioning of N from Alfalfa Silage, Corn Silage, Corn Grain and Soybean Meal into Milk, Urine, and Feces, Using Stable 15N Isotope. *Anim. Feed Sci. Technol.* **2017**, *229*, 91–96, doi:10.1016/j.anifeedsci.2017.05.009.
6. Beckman, J.L.; Weiss, W.P. Nutrient Digestibility of Diets with Different Fiber to Starch Ratios When Fed to Lactating Dairy Cows. *J. Dairy Sci.* **2005**, *88*, 1015–1023, doi:10.3168/jds.S0022-0302(05)72769-7.
7. Benchaar, C.; Hassanat, F.; Gervais, R.; Chouinard, P.Y.; Julien, C.; Petit, H. V.; Massé, D.I. Effects of Increasing Amounts of Corn Dried Distillers Grains with Solubles in Dairy Cow Diets on Methane Production, Ruminal Fermentation, Digestion, N Balance, and Milk Production. *J. Dairy Sci.* **2013**, *96*, 2413–2427, doi:10.3168/jds.2012-6037.
8. Benchaar, C.; Hassanat, F.; Gervais, R.; Chouinard, P.Y.; Petit, H. V.; Massé, D.I. Methane Production, Digestion, Ruminal Fermentation, Nitrogen Balance, and Milk Production of Cows Fed Corn Silage- or Barley Silage-Based Diets. *J. Dairy Sci.* **2014**, *97*, 961–974, doi:10.3168/jds.2013-7122.
9. Benchaar, C.; Hassanat, F.; Martineau, R.; Gervais, R. Linseed Oil Supplementation to Dairy Cows Fed Diets Based on Red Clover Silage or Corn Silage: Effects on Methane Production, Rumen Fermentation, Nutrient Digestibility, N Balance, and Milk Production. *J. Dairy Sci.* **2015**, *98*, 7993–8008, doi:10.3168/jds.2015-9398.
10. Benchaar, C. Feeding Oregano Oil and Its Main Component Carvacrol Does Not Affect Ruminal Fermentation, Nutrient Utilization, Methane Emissions, Milk Production, or Milk Fatty Acid Composition of Dairy Cows. *J. Dairy Sci.* **2020**, *103*, 1516–1527, doi:10.3168/jds.2019-17230.
11. Benchaar, C.; Hassanat, F. Method of Diet Delivery to Dairy Cows: Effects on Nutrient Digestion, Rumen Fermentation, Methane Emissions from Enteric Fermentation and Stored Manure, Nitrogen Excretion, and Milk Production. *J. Dairy Sci.* **2021**, *104*, 11686–11698, doi:10.3168/jds.2021-20466.
12. Benchaar, C. Diet Supplementation with Thyme Oil and Its Main Component Thymol Failed to Favorably Alter Rumen Fermentation, Improve Nutrient Utilization, or Enhance

Milk Production in Dairy Cows. *J. Dairy Sci.* **2021**, *104*, 324–336, doi:10.3168/jds.2020-18401.

13. Borucki Castro, S.I.; Phillip, L.E.; Lapierre, H.; Jardon, P.W.; Berthiaume, R. The Relative Merit of Ruminal Undegradable Protein from Soybean Meal or Soluble Fiber from Beet Pulp to Improve Nitrogen Utilization in Dairy Cows. *J. Dairy Sci.* **2008**, *91*, 3947–3957, doi:10.3168/jds.2007-0638.
14. Brito, A.F.; Broderick, G.A. Effect of Varying Dietary Ratios of Alfalfa Silage to Corn Silage on Production and Nitrogen Utilization in Lactating Dairy Cows. *J. Dairy Sci.* **2006**, *89*, 3924–3938, doi:10.3168/jds.S0022-0302(06)72435-3.
15. Carruthers, V.R.; Neil, P.G. Milk Production and Ruminal Metabolites from Cows Offered Two Pasture Diets Supplemented with Non-Structural Carbohydrate. *New Zeal. J. Agric. Res.* **1997**, *40*, 513–521, doi:10.1080/00288233.1997.9513273.
16. Cheng, L.; Woodward, S.L.; Dewhurst, R.J.; Zhou, H.; Edwards, G.R. Nitrogen Partitioning, Energy Use Efficiency and Isotopic Fractionation Measurements from Cows Differing in Genetic Merit Fed Low-Quality Pasture in Late Lactation. *Anim. Prod. Sci.* **2014**, *54*, 1651–1656, doi:10.1071/AN14171.
17. Cherney, D.J.R.; Cherney, J.H.; Chase, L.E. Influence of Dietary Nonfiber Carbohydrate Concentration and Supplementation of Sucrose on Lactation Performance of Cows Fed Fescue Silage. *J. Dairy Sci.* **2003**, *86*, 3983–3991, doi:10.3168/jds.S0022-0302(03)74009-0.
18. Chibisa, G.E.; Gozho, G.N.; Van Kessel, A.G.; Olkowski, A.A.; Mutsvangwa, T. Effects of Peripartum Propylene Glycol Supplementation on Nitrogen Metabolism, Body Composition, and Gene Expression for the Major Protein Degradation Pathways in Skeletal Muscle in Dairy Cows. *J. Dairy Sci.* **2008**, *91*, 3512–3527, doi:10.3168/jds.2007-0920.
19. Colombini, S.; Galassi, G.; Crovetto, G.M.; Rapetti, L. Milk Production, Nitrogen Balance, and Fiber Digestibility Prediction of Corn, Whole Plant Grain Sorghum, and Forage Sorghum Silages in the Dairy Cow. *J. Dairy Sci.* **2012**, *95*, 4457–4467, doi:10.3168/jds.2011-4444.
20. Eriksson, T.; Murphy, M.; Ciszuk, P.; Burstedt, E. Nitrogen Balance, Microbial Protein Production, and Milk Production in Dairy Cows Fed Fodder Beets and Potatoes, or Barley. *J. Dairy Sci.* **2004**, *87*, 1057–1070, doi:10.3168/jds.S0022-0302(04)73252-X.
21. Flis, S.A.; Wattiaux, M.A. Effects of Parity and Supply of Rumen-Degraded and Undegraded Protein on Production and Nitrogen Balance in Holsteins. *J. Dairy Sci.* **2005**, *88*, 2096–2106, doi:10.3168/jds.S0022-0302(05)72886-1.
22. Foley, A.E.; Hristov, A.N.; Melgar, A.; Ropp, J.K.; Etter, R.P.; Zaman, S.; Hunt, C.W.; Huber, K.; Price, W.J. Effect of Barley and Its Amylopectin Content on Ruminal Fermentation and Nitrogen Utilization in Lactating Dairy Cows. *J. Dairy Sci.* **2006**, *89*, 4321–4335, doi:10.3168/jds.S0022-0302(06)72479-1.
23. Ghelichkhan, M.; Eun, J.S.; Christensen, R.G.; Stott, R.D.; MacAdam, J.W. Urine Volume and Nitrogen Excretion Are Altered by Feeding Birdsfoot Trefoil Compared with Alfalfa in Lactating Dairy Cows. *J. Anim. Sci.* **2018**, *96*, 3993–4001, doi:10.1093/jas/sky259.
24. Haig, P.A.; Mutsvangwa, T.; Spratt, R.; McBride, B.W. Effects of Dietary Protein Solubility on Nitrogen Losses from Lactating Dairy Cows and Comparison with Predictions from the Cornell Net Carbohydrate and Protein System. *J Dairy Sci.* **2002**, *85*,

1208–1217, doi:10.3168/jds.S0022-0302(02)74184-2.
25. Hassanat, F.; Gervais, R.; Julien, C.; Massé, D.I.; Lettat, A.; Chouinard, P.Y.; Petit, H. V.; Benchaar, C. Replacing Alfalfa Silage with Corn Silage in Dairy Cow Diets: Effects on Enteric Methane Production, Ruminal Fermentation, Digestion, N Balance, and Milk Production. *J. Dairy Sci.* **2013**, *96*, 4553–4567, doi:10.3168/jds.2012-6480.
26. Hendriks, S. The Effect of Dietary Nitrogen on Nitrogen Partitioning and Milk Production in Grazing Dairy Cows, Massey University, 2016.
27. Hristov, A.N.; Grandeen, K.L.; Ropp, J.K.; McGuire, M.A. Effect of Sodium Laurate on Ruminal Fermentation and Utilization of Ruminal Ammonia Nitrogen for Milk Protein Synthesis in Dairy Cows. *J. Dairy Sci.* **2004**, *87*, 1820–1831, doi:10.3168/jds.S0022-0302(04)73339-1.
28. Hristov, A.N.; Ropp, J.K. Effect of Dietary Carbohydrate Composition and Availability on Utilization of Ruminal Ammonia Nitrogen for Milk Protein Synthesis in Dairy Cows. *J. Dairy Sci.* **2003**, *86*, 2416–2427, doi:10.3168/jds.S0022-0302(03)73836-3.
29. Hynes, D.N.; Stergiadis, S.; Gordon, A.; Yan, T. Effects of Crude Protein Level in Concentrate Supplements on Animal Performance and Nitrogen Utilization of Lactating Dairy Cows Fed Fresh-Cut Perennial Grass. *J. Dairy Sci.* **2016**, *99*, 8111–8120, doi:10.3168/jds.2016-11110.
30. Ineichen, S.; Marquardt, S.; Wettstein, H.R.; Kreuzer, M.; Reidy, B. Milk Fatty Acid Profile and Nitrogen Utilization of Dairy Cows Fed Ryegrass-Red Clover Silage Containing Plantain (Plantago Lanceolata L.). *Livest. Sci.* **2019**, *221*, 123–132, doi:10.1016/j.livsci.2019.01.018.
31. Judy, J. V.; Bachman, G.C.; Brown-Brandl, T.M.; Fernando, S.C.; Hales, K.E.; Miller, P.S.; Stowell, R.R.; Kononoff, P.J. Energy Balance and Diurnal Variation in Methane Production as Affected by Feeding Frequency in Jersey Cows in Late Lactation. *J. Dairy Sci.* **2018**, *101*, 10899–10910, doi:10.3168/jds.2018-14596.
32. Kauffman, A.J.; St-Pierre, N.R. The Relationship of Milk Urea Nitrogen to Urine Nitrogen Excretion in Holstein and Jersey Cows. *J. Dairy Sci.* **2001**, *84*, 2284–2294, doi:10.3168/jds.s0022-0302(01)74675-9.
33. Kidane, A.; Øverland, M.; Mydland, L.T.; Prestløkken, E. Interaction between Feed Use Efficiency and Level of Dietary Crude Protein on Enteric Methane Emission and Apparent Nitrogen Use Efficiency with Norwegian Red Dairy Cows. *J. Anim. Sci.* **2018**, *96*, 3967–3982, doi:10.1093/jas/sky256.
34. Knowlton, K.F.; Herbein, J.H.; Meister-Weisbarth, M.A.; Wark, W.A. Nitrogen and Phosphorus Partitioning in Lactating Holstein Cows Fed Different Sources of Dietary Protein and Phosphorus. *J. Dairy Sci.* **2001**, *84*, 1210–1217, doi:10.3168/jds.S0022-0302(01)74582-1.
35. Korhonen, M.; Vanhatalo, A.; Huhtanen, P. Effect of Protein Source on Amino Acid Supply, Milk Production, and Metabolism of Plasma Nutrients in Dairy Cows Fed Grass Silage. *J Dairy Sci.* **2002**, *85*, 3336–3351, doi:10.3168/jds.S0022-0302(02)74422-6.
36. Luo, G.; Xu, W.; Yang, J.; Li, Y.; Zhang, L.; Wang, Y.; Lin, C.; Zhang, Y. Effects of Ruminally Degradable Starch Levels on Performance, Nitrogen Balance, and Nutrient Digestibility in Dairy Cows Fed Low Corn-Based Starch Diets. *Asian-Australasian J. Anim. Sci.* **2017**, *30*, 653–659, doi:10.5713/ajas.16.0371.
37. Mackle, T.R.; Parr, C.R.; Bryant, A.M. Nitrogen Fertiliser Effects on Milk Yield and Composition, Pasture Intake, Nitrogen and Energy Partitioning, and Rumen Fermentation

Parameters of Dairy Cows in Early Lactation. *New Zeal. J. Agric. Res.* **1996**, *39*, 341–356, doi:10.1080/00288233.1996.9513194.

38. Marshall, C.J.; Beck, M.R.; Garrett, K.; Barrell, G.K.; Al-Marashdeh, O.; Gregorini, P. Nitrogen Balance of Dairy Cows Divergent for Milk Urea Nitrogen Consuming Either Plantain or Perennial Ryegrass. *Animals* **2021**, *11*, 1–18, doi:10.3390/ani11082464.
39. Minnée, E.M.K.; Leach, C.M.T.; Dalley, D.E. Substituting a Pasture-Based Diet with Plantain (Plantago Lanceolata) Reduces Nitrogen Excreted in Urine from Dairy Cows in Late Lactation. *Livest. Sci.* **2020**, *239*, 104093, doi:10.1016/j.livsci.2020.104093.
40. Miyaji, M.; Matsuyama, H.; Hosoda, K.; Nonaka, K. Effect of Replacing Corn with Brown Rice Grain in a Total Mixed Ration Silage on Milk Production, Ruminal Fermentation and Nitrogen Balance in Lactating Dairy Cows. *Anim. Sci. J.* **2012**, *83*, 585–593, doi:10.1111/j.1740-0929.2011.00996.x.
41. Miyaji, M.; Matsuyama, H.; Hosoda, K. Effect of Substituting Brown Rice for Corn on Lactation and Digestion in Dairy Cows Fed Diets with a High Proportion of Grain. *J. Dairy Sci.* **2014**, *97*, 952–960, doi:10.3168/jds.2013-7046.
42. Morris, D.L.; Brown-Brandl, T.M.; Hales, K.E.; Harvatine, K.J.; Kononoff, P.J. Effects of High-Starch or High-Fat Diets Formulated to Be Isoenergetic on Energy and Nitrogen Partitioning and Utilization in Lactating Jersey Cows. *J. Dairy Sci.* **2020**, *103*, 4378–4389, doi:10.3168/jds.2019-17638.
43. Mutsvangwa, T.; Davies, K.L.; McKinnon, J.J.; Christensen, D.A. Effects of Dietary Crude Protein and Rumen-Degradable Protein Concentrations on Urea Recycling, Nitrogen Balance, Omasal Nutrient Flow, and Milk Production in Dairy Cows. *J. Dairy Sci.* **2016**, *99*, 6298–6310, doi:10.3168/jds.2016-10917.
44. Niu, M.; Appuhamy, J.A.D.R.N.; Leytem, A.B.; Dungan, R.S.; Kebreab, E. Effect of Dietary Crude Protein and Forage Contents on Enteric Methane Emissions and Nitrogen Excretion from Dairy Cows Simultaneously. *Anim. Prod. Sci.* **2016**, *56*, 312–321, doi:10.1071/AN15498.
45. Rius, A.G.; Kittelmann, S.; Macdonald, K.A.; Waghorn, G.C.; Janssen, P.H.; Sikkema, E. Nitrogen Metabolism and Rumen Microbial Enumeration in Lactating Cows with Divergent Residual Feed Intake Fed High-Digestibility Pasture. *J. Dairy Sci.* **2012**, *95*, 5024–5034, doi:10.3168/jds.2012-5392.
46. Ruiz, R.; Tedeschi, L.O.; Marini, J.C.; Fox, D.G.; Pell, A.N.; Jarvis, G.; Russell, J.B. The Effect of a Ruminal Nitrogen (N) Deficiency in Dairy Cows: Evaluation of the Cornell Net Carbohydrate and Protein System Ruminal N Deficiency Adjustment. *J. Dairy Sci.* **2002**, *85*, 2986–2999, doi:10.3168/jds.S0022-0302(02)74384-1.
47. Ruiz, R.; Albrecht, G.L.; Tedeschi, L.O.; Jarvis, G.; Russell, J.B.; Fox, D.G. Effect of Monensin on the Performance and Nitrogen Utilization of Lactating Dairy Cows Consuming Fresh Forage. *J. Dairy Sci.* **2001**, *84*, 1717–1727, doi:10.3168/jds.S0022-0302(01)74607-3.
48. Sannes, R.A.; Messman, M.A.; Vagnoni, D.B. Form of Rumen-Degradable Carbohydrate and Nitrogen on Microbial Protein Synthesis and Protein Efficiency of Dairy Cows. *J. Dairy Sci.* **2002**, *85*, 900–908, doi:10.3168/jds.S0022-0302(02)74148-9.
49. Stelwagen, K.; Beukes, P.C.; Hemmings, C. Effect of Zeolite Administration on Nitrogen Metabolism and Excretion in Lactating Dairy Cows Offered Pasture Herbage. *Anim. Prod. Sci.* **2020**, *61*, 560–567, doi:10.1071/AN19674.
50. Tayyab, U.; Sinclair, L.A.; Wilkinson, R.G.; Humphries, D.J.; Reynolds, C.K. Milk

Production, Rumen Function, and Digestion in Dairy Cows Fed Diets Differing in Predominant Forage and Concentrate Type. *Anim. Feed Sci. Technol.* **2022**, *284*, 115151, doi:10.1016/j.anifeedsci.2021.115151.
51. Wattiaux, M.A.; Karg, K.L. Protein Level for Alfalfa and Corn Silage-Based Diets: II. Nitrogen Balance and Manure Characteristics. *J. Dairy Sci.* **2004**, *87*, 3492–3502, doi:10.3168/jds.S0022-0302(04)73484-0.

R Code used for analysis:

```
library(tidyverse)
library(readxl)
library(DescTools)
library(lme4)
library(lmerTest)
library(chillR)
library(performance)
library(emmeans)

complete.dat <- read_xlsx("data4_10-24-22 update.xlsx", sheet = "All data", col_names = T)
complete.dat$Author.year <- as.factor(complete.dat$Author.year); complete.dat$doi.url <-
as.factor(complete.dat$doi.url); complete.dat$Diet.type <- as.factor(complete.dat$Diet.type)

mean(complete.dat$UN.g.d)

##############################################
##          Assessing Established Models          ##
##############################################

complete.dat$w1 <- as.numeric(1/(complete.dat$UN.SEM^2))
complete.dat$w2 <- as.numeric(complete.dat$w1/mean(complete.dat$w1))

####################
###    Eq 1    ##
####################

complete.dat$Eq.1 <- as.numeric(12.54*complete.dat$MUN.mg.dL) # Jonker 1998
complete.dat$res.Eq.1 <- complete.dat$UN.g.d - complete.dat$Eq.1

RMSE(complete.dat[complete.dat$Diet.type == "TMR",]$UN.g.d,
complete.dat[complete.dat$Diet.type == "TMR",]$Eq.1)
RMSE(complete.dat[complete.dat$Diet.type == "Pasture",]$UN.g.d,
complete.dat[complete.dat$Diet.type == "Pasture",]$Eq.1)
```

```
RMSE(complete.dat[complete.dat$Diet.type == "TMR",]$UN.g.d,
complete.dat[complete.dat$Diet.type ==
"TMR",]$Eq.1)/mean(complete.dat[complete.dat$Diet.type == "TMR",]$UN.g.d)
RMSE(complete.dat[complete.dat$Diet.type == "Pasture",]$UN.g.d,
complete.dat[complete.dat$Diet.type ==
"Pasture",]$Eq.1)/mean(complete.dat[complete.dat$Diet.type == "Pasture",]$UN.g.d)

complete.dat$Eq.1.MC <- as.numeric(complete.dat$Eq.1 - mean(complete.dat$Eq.1))
m1 <- lmer(res.Eq.1 ~ Diet.type*Eq.1.MC + (1|doi.url), weights = w2, data = complete.dat)
summary(m1)
anova(m1)
emmeans(m1, pairwise~Diet.type)
emtrends(m1, pairwise ~ "Diet.type", var = "Eq.2")


###################
###       Eq 2          ##
###################

complete.dat$Eq.2 <- as.numeric(0.0259*complete.dat$MUN.mg.dL*complete.dat$bw.kg) #
Kauffman and St-Pierre, 2001
complete.dat$res.Eq.2 <- complete.dat$UN.g.d - complete.dat$Eq.2



RMSE(complete.dat[complete.dat$Diet.type == "TMR",]$UN.g.d,
complete.dat[complete.dat$Diet.type == "TMR",]$Eq.2)
RMSE(complete.dat[complete.dat$Diet.type == "Pasture",]$UN.g.d,
complete.dat[complete.dat$Diet.type == "Pasture",]$Eq.2)

RMSE(complete.dat[complete.dat$Diet.type == "TMR",]$UN.g.d,
complete.dat[complete.dat$Diet.type ==
"TMR",]$Eq.2)/mean(complete.dat[complete.dat$Diet.type == "TMR",]$UN.g.d)
RMSE(complete.dat[complete.dat$Diet.type == "Pasture",]$UN.g.d,
complete.dat[complete.dat$Diet.type ==
"Pasture",]$Eq.2)/mean(complete.dat[complete.dat$Diet.type == "Pasture",]$UN.g.d)

complete.dat$Eq.2.MC <- as.numeric(complete.dat$Eq.2 - mean(complete.dat$Eq.2))
m1 <- lmer(res.Eq.2 ~ Diet.type*Eq.2.MC + (1|doi.url), weights = w2, data = complete.dat)
summary(m1)
anova(m1)
emmeans(m1, pairwise~Diet.type)
emtrends(m1, pairwise ~ "Diet.type", var = "Eq.2.MC")
```

```
###################
###          Eq 3          ##
###################


complete.dat$Eq.3 <- as.numeric(-
148.8+8.06*complete.dat$MUN.mg.dL+8.91*complete.dat$diet.CP.per+4.06*complete.dat$DM
I) # Spek et al.,2013b
complete.dat$res.Eq.3 <- complete.dat$UN.g.d - complete.dat$Eq.3


RMSE(complete.dat[complete.dat$Diet.type == "TMR",]$UN.g.d,
complete.dat[complete.dat$Diet.type == "TMR",]$Eq.3)
RMSE(complete.dat[complete.dat$Diet.type == "Pasture",]$UN.g.d,
complete.dat[complete.dat$Diet.type == "Pasture",]$Eq.3)

RMSE(complete.dat[complete.dat$Diet.type == "TMR",]$UN.g.d,
complete.dat[complete.dat$Diet.type ==
"TMR",]$Eq.3)/mean(complete.dat[complete.dat$Diet.type == "TMR",]$UN.g.d)
RMSE(complete.dat[complete.dat$Diet.type == "Pasture",]$UN.g.d,
complete.dat[complete.dat$Diet.type ==
"Pasture",]$Eq.3)/mean(complete.dat[complete.dat$Diet.type == "Pasture",]$UN.g.d)

complete.dat$Eq.3.MC <- as.numeric(complete.dat$Eq.3 - mean(complete.dat$Eq.3))
m1 <- lmer(res.Eq.3 ~ Diet.type*Eq.3.MC + (1|doi.url), weights = w2, data = complete.dat)
summary(m1)
anova(m1)
emmeans(m1, pairwise~Diet.type)
emtrends(m1, pairwise ~ "Diet.type", var = "Eq.2.MC")

####################
###        MUN %        ##
####################

m1 <- lmer(res.Eq.1 ~ MUN.mg.dL + (1|doi.url), weights = w2, data = complete.dat)
summary(m1)


m1 <- lmer(res.Eq.2 ~ MUN.mg.dL + (1|doi.url), weights = w2, data = complete.dat)
summary(m1)

m1 <- lmer(res.Eq.3 ~ MUN.mg.dL + (1|doi.url), weights = w2, data = complete.dat)
summary(m1)
```

```
###################
###         CP %       ##
###################

m1 <- lmer(res.Eq.1 ~ diet.CP.per + (1|doi.url), weights = w2, data = complete.dat)
summary(m1)


m1 <- lmer(res.Eq.2 ~ diet.CP.per + (1|doi.url), weights = w2, data = complete.dat)
summary(m1)

m1 <- lmer(res.Eq.3 ~ diet.CP.per + (1|doi.url), weights = w2, data = complete.dat)
summary(m1)

###################
###       BW kg        ##
###################

m1 <- lmer(res.Eq.1 ~ bw.kg + (1|doi.url), weights = w2, data = complete.dat)
summary(m1)

m1 <- lmer(res.Eq.2 ~ bw.kg + (1|doi.url), weights = w2, data = complete.dat)
summary(m1)

m1 <- lmer(res.Eq.3 ~ bw.kg + (1|doi.url), weights = w2, data = complete.dat)
summary(m1)

###################
###         DMI        ##
###################
m1 <- lmer(res.Eq.1 ~ DMI + (1|doi.url), weights = w2, data = complete.dat)
summary(m1)

m1 <- lmer(res.Eq.2 ~ DMI + (1|doi.url), weights = w2, data = complete.dat)
summary(m1)

m1 <- lmer(res.Eq.3 ~ DMI + (1|doi.url), weights = w2, data = complete.dat)
summary(m1)



mean(complete.dat[complete.dat$Diet.type == "TMR",]$diet.CP.per)
mean(complete.dat[complete.dat$Diet.type == "Pasture",]$diet.CP.per)

mean(complete.dat[complete.dat$Diet.type == "TMR",]$Diet.NDF.per, na.rm =T)
mean(complete.dat[complete.dat$Diet.type == "Pasture",]$Diet.NDF.per)
```

```
mean(complete.dat[complete.dat$Diet.type == "TMR",]$per.forage.of.diet, na.rm = T)
mean(complete.dat[complete.dat$Diet.type == "Pasture",]$per.forage.of.diet)

mean(complete.dat[complete.dat$Diet.type == "TMR",]$UN.g.d)
mean(complete.dat[complete.dat$Diet.type == "Pasture",]$UN.g.d)

mean(complete.dat[complete.dat$Diet.type == "TMR",]$MUN.mg.dL)
mean(complete.dat[complete.dat$Diet.type == "Pasture",]$MUN.mg.dL)

mean(complete.dat[complete.dat$Diet.type == "TMR",]$bw.kg)
mean(complete.dat[complete.dat$Diet.type == "Pasture",]$bw.kg)

mean(complete.dat[complete.dat$Diet.type == "TMR",]$DMI)
mean(complete.dat[complete.dat$Diet.type == "Pasture",]$DMI)

mean(complete.dat[complete.dat$Diet.type == "TMR",]$N.int.g.d)
mean(complete.dat[complete.dat$Diet.type == "Pasture",]$N.int.g.d)

mean(complete.dat[complete.dat$Diet.type == "TMR",]$MY)
mean(complete.dat[complete.dat$Diet.type == "Pasture",]$MY)

mean(complete.dat[complete.dat$Diet.type == "TMR",]$DIM, na.rm = T)
mean(complete.dat[complete.dat$Diet.type == "Pasture",]$DIM, na.rm = T)


sd(complete.dat[complete.dat$Diet.type == "TMR",]$diet.CP.per, na.rm = T)
sd(complete.dat[complete.dat$Diet.type == "Pasture",]$diet.CP.per)

sd(complete.dat[complete.dat$Diet.type == "TMR",]$Diet.NDF.per)
sd(complete.dat[complete.dat$Diet.type == "Pasture",]$Diet.NDF.per)

sd(complete.dat[complete.dat$Diet.type == "TMR",]$per.forage.of.diet, na.rm = T)
sd(complete.dat[complete.dat$Diet.type == "Pasture",]$per.forage.of.diet)

sd(complete.dat[complete.dat$Diet.type == "TMR",]$UN.g.d)
sd(complete.dat[complete.dat$Diet.type == "Pasture",]$UN.g.d)

sd(complete.dat[complete.dat$Diet.type == "TMR",]$MUN.mg.dL)
sd(complete.dat[complete.dat$Diet.type == "Pasture",]$MUN.mg.dL)

sd(complete.dat[complete.dat$Diet.type == "TMR",]$bw.kg)
sd(complete.dat[complete.dat$Diet.type == "Pasture",]$bw.kg)

sd(complete.dat[complete.dat$Diet.type == "TMR",]$DMI)
sd(complete.dat[complete.dat$Diet.type == "Pasture",]$DMI)
```

```
sd(complete.dat[complete.dat$Diet.type == "TMR",]$N.int.g.d)
sd(complete.dat[complete.dat$Diet.type == "Pasture",]$N.int.g.d)

sd(complete.dat[complete.dat$Diet.type == "TMR",]$MY)
sd(complete.dat[complete.dat$Diet.type == "Pasture",]$MY)

sd(complete.dat[complete.dat$Diet.type == "TMR",]$DIM, na.rm = T)
sd(complete.dat[complete.dat$Diet.type == "Pasture",]$DIM)


########################################
##              Model Development             ##
########################################

MUNvUN <- read_xlsx("data4_10-24-22 update.xlsx", sheet = "Training", col_names = T)
MUNvUN$Author.year <- as.factor(MUNvUN$Author.year); MUNvUN$doi.url <-
as.factor(MUNvUN$doi.url); MUNvUN$Diet.type <- as.factor(MUNvUN$Diet.type)


MUNvUN$W1 <- as.numeric(1/(MUNvUN$UN.BW.SEM^2))
MUNvUN$W2 <- as.numeric(MUNvUN$W1/mean(MUNvUN$W1))

## Eq. 4 Developement

this.lm <- lmer(UN.g.bw ~ -1+MUN.mg.dL:Diet.type + (1|doi.url), weights = W2, data =
MUNvUN)
summary(this.lm)
anova(this.lm)
r2_nakagawa(this.lm)


## Eq. 5 Development

this.lm <- lmer(UN.g.d ~ MUN.mg.dL + diet.CP.per + Diet.NDF.per + DMI +(1|doi.url),
weights = W2, data = MUNvUN)
summary(this.lm)
anova(this.lm)


## Eq. 6 Development

this.lm <- lmer(UN.g.d ~ MUN.mg.dL + diet.CP.per + Diet.NDF.per + DMI + bw.kg
+(1|doi.url), weights = W2, data = MUNvUN)
summary(this.lm)
anova(this.lm)
```

```
## Eq. 7 Development

this.lm <- lmer(UN.g.bw ~ MUN.mg.dL + diet.CP.per + (1|doi.url), weights = W2, data =
MUNvUN)
summary(this.lm)
anova(this.lm)



this.lm <- lmer(UN.g.d ~ bw.kg + MUN.mg.dL:bw.kg + diet.CP.per:bw.kg + (1|doi.url), weights
= W2, data = MUNvUN)
summary(this.lm)
anova(this.lm)

#################################
##              Descriptive Stats           ##
#################################


mean(MUNvUN[MUNvUN$Diet.type == "TMR",]$diet.CP.per)
mean(MUNvUN[MUNvUN$Diet.type == "Pasture",]$diet.CP.per)

mean(MUNvUN[MUNvUN$Diet.type == "TMR",]$Diet.NDF.per, na.rm=T)
mean(MUNvUN[MUNvUN$Diet.type == "Pasture",]$Diet.NDF.per)

mean(MUNvUN[MUNvUN$Diet.type == "TMR",]$per.forage.of.diet, na.rm = T)
mean(MUNvUN[MUNvUN$Diet.type == "Pasture",]$per.forage.of.diet)

mean(MUNvUN[MUNvUN$Diet.type == "TMR",]$UN.g.d)
mean(MUNvUN[MUNvUN$Diet.type == "Pasture",]$UN.g.d)

mean(MUNvUN[MUNvUN$Diet.type == "TMR",]$MUN.mg.dL)
mean(MUNvUN[MUNvUN$Diet.type == "Pasture",]$MUN.mg.dL)

mean(MUNvUN[MUNvUN$Diet.type == "TMR",]$bw.kg)
mean(MUNvUN[MUNvUN$Diet.type == "Pasture",]$bw.kg)

mean(MUNvUN[MUNvUN$Diet.type == "TMR",]$DMI)
mean(MUNvUN[MUNvUN$Diet.type == "Pasture",]$DMI)

mean(MUNvUN[MUNvUN$Diet.type == "TMR",]$N.int.g.d)
mean(MUNvUN[MUNvUN$Diet.type == "Pasture",]$N.int.g.d)

mean(MUNvUN[MUNvUN$Diet.type == "TMR",]$MY)
mean(MUNvUN[MUNvUN$Diet.type == "Pasture",]$MY)
```

```
mean(MUNvUN[MUNvUN$Diet.type == "TMR",]$DIM, na.rm = T)
mean(MUNvUN[MUNvUN$Diet.type == "Pasture",]$DIM, na.rm = T)



sd(MUNvUN[MUNvUN$Diet.type == "TMR",]$diet.CP.per)
sd(MUNvUN[MUNvUN$Diet.type == "Pasture",]$diet.CP.per)

sd(MUNvUN[MUNvUN$Diet.type == "TMR",]$Diet.NDF.per, na.rm = T)
sd(MUNvUN[MUNvUN$Diet.type == "Pasture",]$Diet.NDF.per)

sd(MUNvUN[MUNvUN$Diet.type == "TMR",]$per.forage.of.diet, na.rm = T)
sd(MUNvUN[MUNvUN$Diet.type == "Pasture",]$per.forage.of.diet)

sd(MUNvUN[MUNvUN$Diet.type == "TMR",]$UN.g.d)
sd(MUNvUN[MUNvUN$Diet.type == "Pasture",]$UN.g.d)

sd(MUNvUN[MUNvUN$Diet.type == "TMR",]$MUN.mg.dL)
sd(MUNvUN[MUNvUN$Diet.type == "Pasture",]$MUN.mg.dL)

sd(MUNvUN[MUNvUN$Diet.type == "TMR",]$bw.kg)
sd(MUNvUN[MUNvUN$Diet.type == "Pasture",]$bw.kg)

sd(MUNvUN[MUNvUN$Diet.type == "TMR",]$DMI)
sd(MUNvUN[MUNvUN$Diet.type == "Pasture",]$DMI)

sd(MUNvUN[MUNvUN$Diet.type == "TMR",]$N.int.g.d)
sd(MUNvUN[MUNvUN$Diet.type == "Pasture",]$N.int.g.d)

sd(MUNvUN[MUNvUN$Diet.type == "TMR",]$MY)
sd(MUNvUN[MUNvUN$Diet.type == "Pasture",]$MY)

sd(MUNvUN[MUNvUN$Diet.type == "TMR",]$DIM, na.rm = T)
sd(MUNvUN[MUNvUN$Diet.type == "Pasture",]$DIM)


##################################################
##          Model validation and comparison TMR        ##
##################################################

test <- read_xlsx("data4_10-24-22 update.xlsx", sheet = "Test set")


mean(test[test$Diet.type == "TMR",]$diet.CP.per)
mean(test[test$Diet.type == "Pasture",]$diet.CP.per)
```

```
mean(test[test$Diet.type == "TMR",]$Diet.NDF.per)
mean(test[test$Diet.type == "Pasture",]$Diet.NDF.per)

mean(test[test$Diet.type == "TMR",]$per.forage.of.diet, na.rm = T)
mean(test[test$Diet.type == "Pasture",]$per.forage.of.diet)

mean(test[test$Diet.type == "TMR",]$UN.g.d)
mean(test[test$Diet.type == "Pasture",]$UN.g.d)

mean(test[test$Diet.type == "TMR",]$MUN.mg.dL)
mean(test[test$Diet.type == "Pasture",]$MUN.mg.dL)

mean(test[test$Diet.type == "TMR",]$bw.kg)
mean(test[test$Diet.type == "Pasture",]$bw.kg)

mean(test[test$Diet.type == "TMR",]$DMI)
mean(test[test$Diet.type == "Pasture",]$DMI)

mean(test[test$Diet.type == "TMR",]$N.int.g.d)
mean(test[test$Diet.type == "Pasture",]$N.int.g.d)

mean(test[test$Diet.type == "TMR",]$MY)
mean(test[test$Diet.type == "Pasture",]$MY)

mean(test[test$Diet.type == "TMR",]$DIM)
mean(test[test$Diet.type == "Pasture",]$DIM, na.rm = T)


sd(test[test$Diet.type == "TMR",]$diet.CP.per)
sd(test[test$Diet.type == "Pasture",]$diet.CP.per)

sd(test[test$Diet.type == "TMR",]$Diet.NDF.per)
sd(test[test$Diet.type == "Pasture",]$Diet.NDF.per)

sd(test[test$Diet.type == "TMR",]$per.forage.of.diet, na.rm = T)
sd(test[test$Diet.type == "Pasture",]$per.forage.of.diet)

sd(test[test$Diet.type == "TMR",]$UN.g.d)
sd(test[test$Diet.type == "Pasture",]$UN.g.d)

sd(test[test$Diet.type == "TMR",]$MUN.mg.dL)
sd(test[test$Diet.type == "Pasture",]$MUN.mg.dL)

sd(test[test$Diet.type == "TMR",]$bw.kg)
sd(test[test$Diet.type == "Pasture",]$bw.kg)
```

```
sd(test[test$Diet.type == "TMR",]$DMI)
sd(test[test$Diet.type == "Pasture",]$DMI)

sd(test[test$Diet.type == "TMR",]$N.int.g.d)
sd(test[test$Diet.type == "Pasture",]$N.int.g.d)

sd(test[test$Diet.type == "TMR",]$MY)
sd(test[test$Diet.type == "Pasture",]$MY)

sd(test[test$Diet.type == "TMR",]$DIM)
sd(test[test$Diet.type == "Pasture",]$DIM)

test$Eq.4 <- as.numeric(test$bw.kg*test$MUN.mg.dL*(0.0214+0.0026*test$DT))

test$Eq.5 <- as.numeric(-209.4+6.8*test$MUN.mg.dL+12.4*test$diet.CP.per+4.8*test$DMI)

test$Eq.6 <- as.numeric(-281.3+6.7*test$MUN.mg.dL+13.2*test$diet.CP.per+2.8*test$DMI +
0.16*test$bw.kg)

test$Eq.7 <- as.numeric(-0.253*test$bw.kg + test$bw.kg*test$MUN.mg.dL*0.00932 +
test$bw.kg*test$diet.CP.per*0.026)

#Eq.4
CCC(test$UN.g.d, test$Eq.4)
cor.test(test$UN.g.d, test$Eq.4)
RMSEP(test$UN.g.d, test$Eq.4)
RMSEP(test$UN.g.d, test$Eq.4)/mean(test$UN.g.d)

#Eq.5
CCC(test$UN.g.d, test$Eq.5)
cor.test(test$UN.g.d, test$Eq.5)
RMSEP(test$UN.g.d, test$Eq.5)
RMSEP(test$UN.g.d, test$Eq.5)/mean(test$UN.g.d)

#Eq.6
CCC(test$UN.g.d, test$Eq.6)
cor.test(test$UN.g.d, test$Eq.6)
RMSEP(test$UN.g.d, test$Eq.6)
RMSEP(test$UN.g.d, test$Eq.6)/mean(test$UN.g.d)

#Eq.7
CCC(test$UN.g.d, test$Eq.7)
cor.test(test$UN.g.d, test$Eq.7)
RMSEP(test$UN.g.d, test$Eq.7)
RMSEP(test$UN.g.d, test$Eq.7)/mean(test$UN.g.d)
```

```
mean(test$UN.g.d)
mean(test$Eq.4)
mean(test$Eq.5)
mean(test$Eq.6)
mean(test$Eq.7)

sd(test$UN.g.d)
sd(test$Eq.4)
sd(test$Eq.5)
sd(test$Eq.6)
sd(test$Eq.7)

sd(test$UN.g.d)/mean(test$UN.g.d)
sd(test$Eq.4)/mean(test$Eq.4)
sd(test$Eq.5)/mean(test$Eq.5)
sd(test$Eq.6)/mean(test$Eq.6)
sd(test$Eq.7)/mean(test$Eq.7)

max(test$UN.g.d)
max(test$Eq.4)
max(test$Eq.5)
max(test$Eq.6)
max(test$Eq.7)

min(test$UN.g.d)
min(test$Eq.4)
min(test$Eq.5)
min(test$Eq.6)
min(test$Eq.7)

##################################
##              Figure Plotting              ##
##################################

mod.plot <- subset(test, select = c("UN.g.d","Eq.4", "Eq.5", "Eq.6", "Eq.7"))
names(mod.plot) <- c("Actual","Eq. 4", "Eq. 5", "Eq. 6", "Eq. 7")
mod.plot.long <- mod.plot %>% gather(key = "Models", value = "Predicted UN, g/d", 2:5)

mod.plot.long$Models <- factor(mod.plot.long$Models, levels = c("Eq. 4", "Eq. 5", "Eq. 6",
"Eq. 7"))

ggplot(mod.plot.long, aes(x = Actual, y = `Predicted UN, g/d`)) + geom_smooth(method = lm,
formula = y~x, color = "black", size = 1) + labs(x = "Actual UN, g/d")  + geom_abline(slope = 1,
intercept = 0, size = 0.5) + scale_linetype_manual(values=c(1, 2, 3, 4)) + geom_point() +
theme_classic() + facet_grid(.~Models) + ggsave("Figure 2.jpeg", height = 4, width = 6, dpi =
1200)
```
